# Interrogation of novel CDK2/9 inhibitor fadraciclib (CYC065) as a potential therapeutic approach for AML

**DOI:** 10.1038/s41420-021-00496-y

**Published:** 2021-06-10

**Authors:** Wittawat Chantkran, Ya-Ching Hsieh, Daniella Zheleva, Sheelagh Frame, Helen Wheadon, Mhairi Copland

**Affiliations:** 1 Paul O’Gorman Leukaemia Research Centre, Institute of Cancer Sciences, College of Medical, Veterinary and Life Sciences, University of Glasgow, Glasgow, UK; 2 Department of Pathology, Phramongkutklao College of Medicine, Bangkok, Thailand; 3 Cyclacel Limited, Dundee, UK

**Keywords:** Acute myeloid leukaemia, Preclinical research

## Abstract

Over the last 50 years, there has been a steady improvement in the treatment outcome of acute myeloid leukemia (AML). However, median survival in the elderly is still poor due to intolerance to intensive chemotherapy and higher numbers of patients with adverse cytogenetics. Fadraciclib (CYC065), a novel cyclin-dependent kinase (CDK) 2/9 inhibitor, has preclinical efficacy in AML. In AML cell lines, myeloid cell leukemia 1 (MCL-1) was downregulated following treatment with fadraciclib, resulting in a rapid induction of apoptosis. In addition, RNA polymerase II (RNAPII)-driven transcription was suppressed, rendering a global gene suppression. Rapid induction of apoptosis was observed in primary AML cells after treatment with fadraciclib for 6–8 h. Twenty-four hours continuous treatment further increased efficacy of fadraciclib. Although preliminary results showed that AML cell lines harboring *KMT2A* rearrangement (*KMT2A*-r) are more sensitive to fadraciclib, we found that the drug can induce apoptosis and decrease MCL-1 expression in primary AML cells, regardless of *KMT2A* status. Importantly, the diversity of genetic mutations observed in primary AML patient samples was associated with variable response to fadraciclib, confirming the need for patient stratification to enable a more effective and personalized treatment approach. Synergistic activity was demonstrated when fadraciclib was combined with the BCL-2 inhibitor venetoclax, or the conventional chemotherapy agents, cytarabine, or azacitidine, with the combination of fadraciclib and azacitidine having the most favorable therapeutic window. In summary, these results highlight the potential of fadraciclib as a novel therapeutic approach for AML.

## Introduction

Acute myeloid leukemia (AML) is one of the most common hematologic malignancies, characterized by clonal, proliferative, and abnormally or poorly differentiated myeloid cells infiltrating the bone marrow, blood or extramedullary tissues^1–3^. Although treatment has progressed, in some AML patients, particularly the elderly^1,4^, outcome is dismal due to the remarkable genetic complexity^5,6^, epigenetic alterations^7–9^, and the dynamics of the disease^10,11^. Treatment-related morbidity/mortality and resistance to chemotherapy are major causes of treatment failure^12^. However, longer life expectancy and possibly a cure is achievable if complete remission (CR) is attained^13,14^. Therefore, there is substantial research to improve and personalize therapies, and lessen treatment toxicity^15–18^.

Currently, several novel agents acting through distinct molecular targets, identified in the pathophysiology of AML, are being investigated alone or combined with conventional chemotherapy, e.g., FLT3 inhibitors (midostaurin^19^, gilteritinib^20^), IDH1/2 inhibitors (ivosidenib^21^, enasidenib^22,23^), BCL-2 inhibitor (venetoclax^24,25^), and cyclin-dependent kinase (CDK) inhibitors (CDKi; alvocidib^26–28^, fadraciclib^29^), which cause cell cycle arrest and induce apoptosis.

The second generation CDKi fadraciclib is an orally available 2,6,9-trisubstituted purine analog designed to selectively inhibit CDK2 and CDK9^29–31^. Direct inhibition of CDK2 results in cell cycle arrest of abnormally proliferating cells^32^. The blockade of CDK9 halts RNA polymerase II (RNAPII) transcriptional activity consequently inducing apoptosis^33^. Furthermore, CDK9 inhibition downregulates the anti-apoptotic protein myeloid cell leukemia 1 (MCL-1)^34^. Fadraciclib has already entered clinical trials in advanced solid tumors (NCT02552953), chronic lymphocytic leukemia (CLL) (NCT03739554) and AML (NCT04017546).

In this study, fadraciclib was assessed for efficacy against AML cell lines and primary AML samples in vitro either as a single agent or in combination with venetoclax (VEN), cytarabine (AraC), or azacitidine (AZA).

## Results

### Fadraciclib induces apoptosis of AML cell lines and reduces metabolic activity confirming anti-proliferative effect

**Fig. 1:**
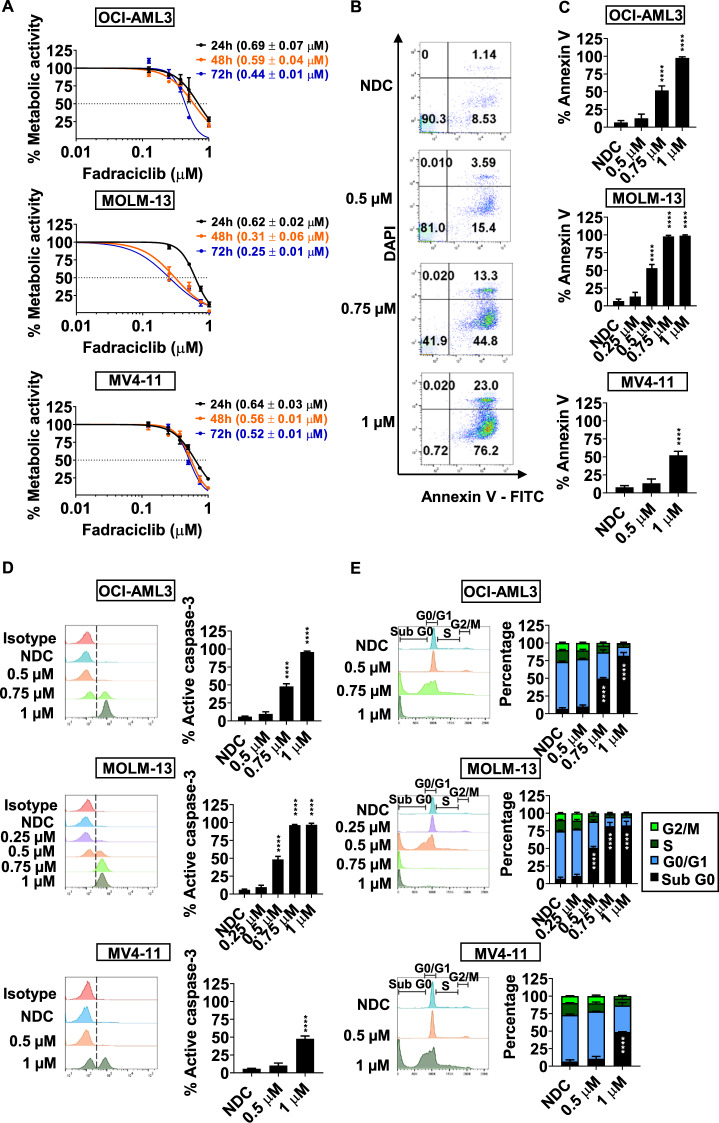
Fadraciclib reduces metabolic activity and causes apoptosis of AML cell lines. **A** The IC50 of fadraciclib at 24, 48 and 72 h in AML cell lines using the rezasurin reduction assay. **B** Representative flow cytometry plots Annexin V/DAPI apoptosis assay for the OCI-AML3 cell line treated with fadraciclib for 72 h. **C** Bar charts based on flow cytometry data for the percentage of annexin V-positive cells in AML cell lines treated with fadraciclib for 72 h. **D** Representative flow cytometry plots and summary bar charts of the percentage of active caspase-3-positive cells in AML cell lines treated with fadraciclib for 72 h. **E** Representative flow cytometry plots and summary bar charts of cell cycle phase using PI staining of AML cell lines treated with fadraciclib for 72 h. *n* = 3.Graphs depict mean ± SD (****p < 0.0001). Data were compared using ANOVA.

IC50 of fadraciclib was established by resazurin reduction assay at 24, 48, and 72 h. The IC50 of fadraciclib was lower at 72 h, compared to 24 and 48 h, showing that longer exposure increased drug potency (Fig. 1A). At 72 h, the IC50 of fadraciclib in OCI-AML3, MOLM-13 and MV4–11 was approximately 0.44 ± 0.01 µM, 0.25 ± 0.01 µM and 0.52 ± 0.01 µM, respectively. As compared to the IC50, which is related to anti-proliferative activity, higher fadraciclib doses were required to induce apoptosis in 50% of cells, represented by the percentage of annexin V-positive cells (%annexinV). 0.75, 0.5, and 1 µM of fadraciclib was required to induce apoptosis in 50% of cells in OCI-AML3, MOLM-13 and MV4–11, respectively (Fig. 1B, C). This was confirmed by a significant concentration-dependent increase in the percentage of active caspase-3-positive cells (%active caspase-3; Fig. 1D). Cell cycle analysis at 72 h demonstrated a significant increase in the percentage of sub G0 (%subG0) population at concentrations of 0.75 μM, 0.5 μM, and 1 μM of fadraciclib for OCI-AML3, MOLM-13, and MV4–11, respectively, with no evidence of cell cycle arrest (Fig. 1E). However, at 4 h, G1 arrest occurred in OCI-AML3 and MV4–11, but not MOLM-13 (Supplementary Fig. S1).

Taken together, fadraciclib potently induces apoptosis, increases the subG0 population, and reduces proliferation as evidenced by reduced metabolic activity in AML cell lines.

### Fadraciclib downregulates MCL-1, resulting in rapid induction of apoptosis in AML cell lines

**Fig. 2:**
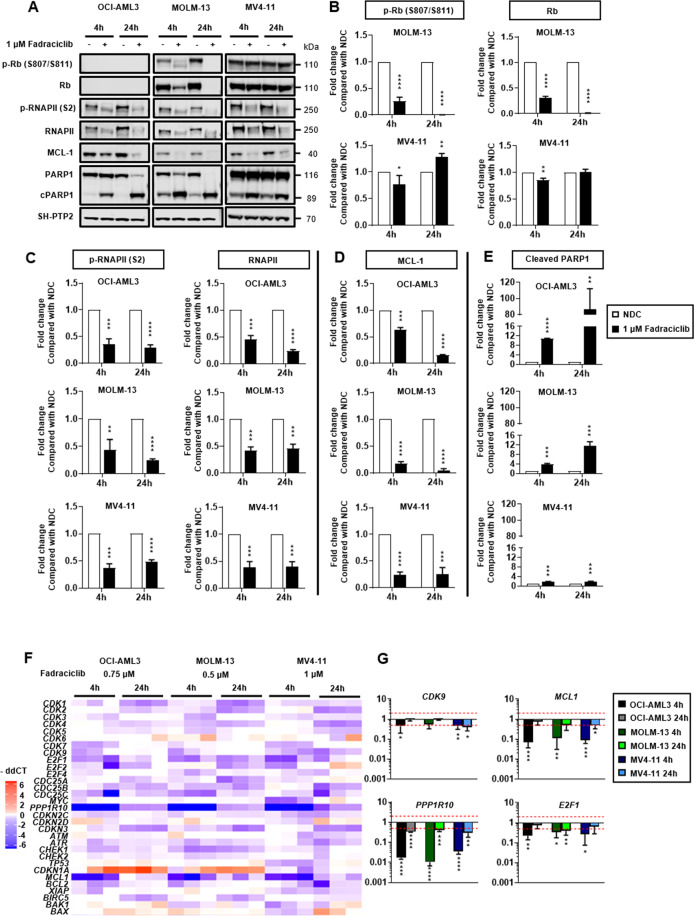
Fadraciclib downregulates MCL-1, resulting in rapid induction of apoptosis in lines treated with 1 µM of fadraciclib for 4 and 24 h. SH-PTP2 was used as an internal AML cell lines. **A** Representative Western blot of OCI-AML3, MOLM-13, and MV4-11 cell protein loading control. **B** Densitometric analysis of serine 807/serine 811 (S807/S811) phosphorylated and total Rb. **C** Densitometric analysis of serine 2 (S2) phosphorylated RNAPII and total RNAPII. **D** Densitometric analysis of MCL-1. **E** Densitometric analysis of cleaved poly (ADP-ribose) polymerase-1 (PARP1). **F** Heatmap demonstrating fold changes relative to NDC of gene expression of OCI-AML3, MOLM-13, and MV4-11 cell lines treated with 0.75, 0.5, and 1 µM of fadraciclib, respectively, for 4 and 24 h. **G** Fold changes of *CDK9*, *MCL1*, *PPP1R10*, and *E2F1* gene expression as compared with NDC. *n* = 3. Graphs depict mean ± SD (*p < 0.05, **p < 0.01, ***p < 0.001, ****p < 0.0001). Data were compared using the Student’s *t*-test.

AML cell lines were treated with 1 µM fadraciclib, for 4 and 24 h. An inhibitory effect on downstream targets was demonstrated by immunoblotting (Fig. 2A). Inhibition of CDK2 and CDK9 was evaluated by phosphorylation levels at serine 807/serine 811 of retinoblastoma (Rb) and serine 2 of RNAPII, respectively. Rb was not expressed in OCI-AML3. In MOLM-13, densitometry confirmed a 75% reduction in Rb levels by 4 h (*p* < 0.0001) and greater than 99% at 24 h, with a corresponding reduction in the phosphorylated form (p-Rb). In MV4–11, only a modest reduction in the levels of Rb and p-Rb at 4 h, with Rb level returning to baseline and a modest increase in p-Rb observed at 24 h (Fig. 2A, B). At 4 h, in all cell lines, the level of RNAPII decreased by approximately 60% with a corresponding decrease in phosphorylation (Fig. 2A, C); with similar results at 24 h.

A significant downregulation of MCL-1 occurred at 4 h in all cell lines and was maintained or further decreased at 24 h (Fig. 2A, D). This induced apoptosis as indicated by the accumulation of cleaved poly (ADP-ribose) polymerase-1 (Fig. 2A, E). The function of CDK1 was not perturbed as observed by an unchanged level of threonine 320 phosphorylated protein phosphatase 1 alpha (Supplementary Fig. S2A, B).

Owing to the impact of fadraciclib on MCL-1 levels, relevant signaling pathways, controlling MCL-1 were investigated. Threonine 180/tyrosine 182 phosphorylated p38 MAPK was markedly increased at 4 h in MOLM-13 (*p* < 0.0001) and MV4–11 (*p* < 0.001), and at 24 h in MOLM-13, but not in OCI-AML3 (Supplementary Fig. S2A, C). A decrease in threonine 202/tyrosine 204 phosphorylated Erk1/2 was also observed at 4 and 24 h (Supplementary Fig. S2A, D). By contrast, serine 473 phosphorylated Akt (Supplementary Fig. S2A, E) and serine 9 phosphorylated GSK3β (Supplementary Fig. S2A, F) were not significantly changed.

These results imply that stress response and apoptosis are potently induced in MOLM-13 and MV4–11^35–38^, and the observed downregulation of MCL-1 was unlikely to be via the proteasome-dependent degradation pathway modulated by GSK3β^39^.

### Fadraciclib alters cell cycle regulatory gene expression to induce apoptosis

OCI-AML3, MOLM-13 and MV4–11 were treated with 0.75, 0.5, and 1 µM of fadraciclib, respectively, for 4 and 24 h and the effect on cell cycle regulatory gene expression investigated. The heatmap depicts fold change of gene expression as compared with NDC (Fig. 2F). Several target genes were downregulated following fadraciclib treatment, including; CDK family, transcription factors, key protein phosphatases, CDKI, DNA damage response regulators, anti-apoptotic related, and pro-apoptotic related genes (Supplementary Figs. S3–S5).

In particular, at 4 h, the expression of CDKs, especially *CDK9*, the transcription factor *E2F1*, and *MCL1*, were suppressed. *PPP1R10* was also significantly downregulated (Fig. 2F, G). Effects on gene expression were less marked at 24 h. Conversely, the DNA damage response regulator gene *CDKN1A*, encoding p21^Cip1^, was upregulated at 24 h, indicating the intrinsic apoptosis pathway^40^ was activated following fadraciclib treatment (Supplementary Fig. S4B).

### Fadraciclib induces apoptosis and decreases MCL-1 expression in primary AML cells, regardless of *KMT2A*-PTD status

Published results indicate AML cell lines bearing *KMT2A* rearrangements (*KMT2A*-r) are sensitive to fadraciclib^29^. Five primary AML samples harboring *KMT2A*-partial tandem duplication (PTD) and five primary AML samples with *KMT2A* wild-type (WT) were tested with fadraciclib as a single agent (Table. 1). All samples were treated with 0.5 and 1 µM fadraciclib for 24 h, then the drug was washed out and cells were cultured in fresh media for up to 72 h.

**Table. 1:** Demographic data of AML patients with their cytogenetic abnormalities and genetic lesions classified by functions.

Sample	Sex	Age (years)	Karyotype	Genetic lesions						
				Signaling genes	DNA methylation-related genes	Chromatin-modifying genes	Nucleophosmin gene	Cohesin complex genes	Spliceosome complex genes	Transcription factor fusions
An investigation of the effect of fadraciclib on primary AML cells harboring *KMT2A*-PTD or *KMT2A* WT										
AML 22	M	68	Normal	*FLT3*-ITD	*DNMT3A*, *TET2*	*KMT2A*-PTD			*SRSF2*	*RUNX1*
AML 20B	M	80	NA			*KMT2A*-PTD				*U2AF1*
AML H002	NA	NA	Normal		*IDH2*	*KMT2A*-PTD, *ASXL1*				
AML 12	F	65	Normal		*DNMT3A*	*KMT2A*-PTD				
AML 17	M	72	Trisomy 21	*NRAS*		*KMT2A*-PTD, *ASXL1*	*NPM1*			*U2F1*
AML 18	M	68	Normal	*NRAS*	*TET2*	*ASXL1*, *EZH2*				
AML 13	F	43	Normal	*FLT3*-ITD			*NPM1*			
AML 44	M	79	Monosomy 7	*FLT3*-ITD	*DNMT3A, IDH2*					
AML 5	M	46	Trisomy 8 t(15;17)		*TET2*	*ASXL1*			*SRSF2*	
AML 36	M	78	Trisomy 11		*DNMT3A*, *IDH1*			*STAG2*	*SRSF2*	
Drug combination studies										
AML H002	NA	NA	Normal		*IDH2*	*KMT2A*-PTD, *ASXL1*				
AML 44	M	79	Monosomy 7	*FLT3*-ITD	*DNMT3A*, *IDH2*					
AML 52	M	73	Normal	*FLT3*-ITD	*DNMT3*		*NPM1*	*RAD21*		
AML 31	F	56	Complex	*KIT*			*NPM1*			
AML 36	M	78	Trisomy 11		*DNMT3A*, *IDH1*			*STAG2*	*SRSF2*	
AML 22	M	68	Normal	*FLT3*-ITD	*DNMT3A TET2*	*KMT2A*-PTD			*SRSF2*	*RUNX1*

**Fig. 3:**
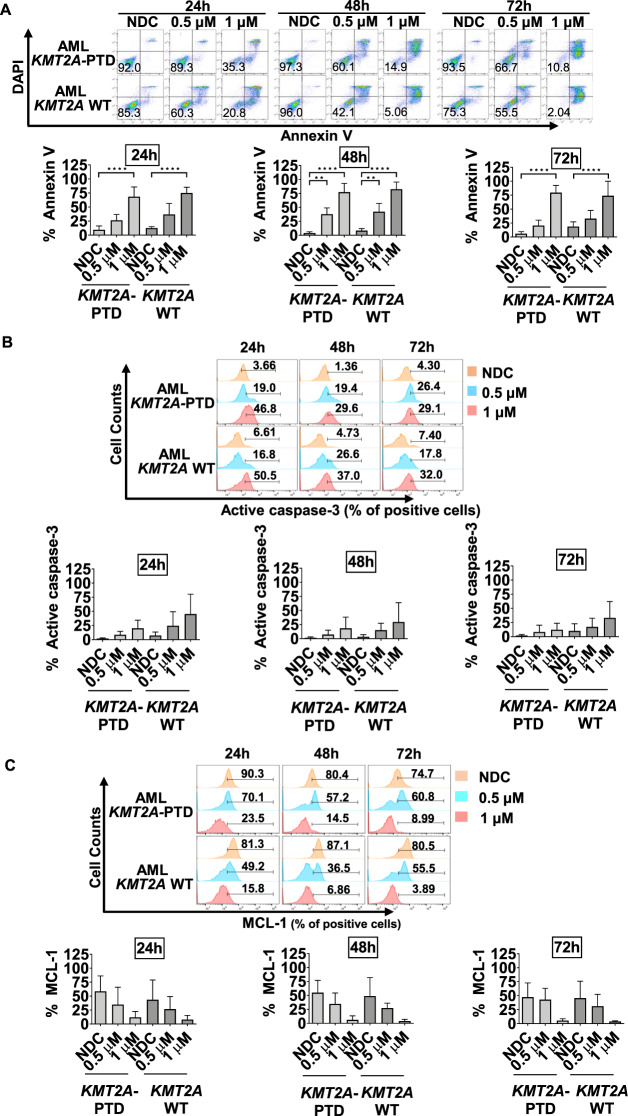
Fadraciclib induces apoptosis and decreased MCL-1 expression in primary AML cells, regardless of *KMT2A*-PTD status. All samples were treated with 0.5 µM or 1 µM of fadraciclib for 24 h, then the drug was washed out and cells were cultured in fresh media for up to 72 h. **A** Representative flow cytometry plots and summary bar charts of the percentage of annexin V-positive cells. *n* = 4–5. **B** Representative flow cytometry plots and summary bar charts of the percentage of active caspase-3-positive cells. *n* = 4–5. **C** Representative flow cytometry plots and summary bar charts of the percentage of the MCL-1 level. *n* = 3. Graphs depict mean ± SD (**p < 0.01, ****p < 0.0001). Data were compared using ANOVA.

At 24, 48, and 72 h time points, fadraciclib treatment resulted in an increase in apoptosis in both *KMT2A*-PTD and *KMT2A* WT patient samples (Fig. 3A, B). The proportion of apoptotic cells significantly increased over time. Specifically, at 72 h, a concentration-dependent maximal increase in %annexinV was observed in *KMT2A*-PTD patient samples following treatment with fadraciclib at 0.5 µM (20.5 ± 9.6%, *p* = 0.6051) and 1 µM (79.5 ± 12.9%, *p* < 0.0001), and in *KMT2A* WT patient samples following treatment with fadraciclib at 0.5 µM (33 ± 14.6%, *p* = 0.624) and 1 µM (74 ± 26%, *p* < 0.0001) compared with NDC (Fig. 3A). Responses were similar in *KMT2A*-PTD and *KMT2A* WT patient samples.

A concentration-dependent increase in %active caspase-3 was observed in both *KMT2A*-PTD and *KMT2A* WT patient samples following fadraciclib treatment (Fig. 3B). However, due to genetic heterogeneity of AML samples (Table. 1), this difference did not reach statistical significance.

Treatment with fadraciclib resulted in a time- and concentration-dependent decrease in MCL-1 expression in both *KMT2A*-PTD and WT AML samples. Specifically, at 72 h, a decrease in MCL-1 in *KMT2A*-PTD patient samples at 0.5 µM (42.8 ± 20.3%, *p* = 0.9997) and 1 µM (5.4 ± 3.2%, *p* = 0.1852), and in *KMT2A* WT patient samples at 0.5 µM (31.2 ± 21.2%, *p* = 0.9463) and 1 µM (4 ± 1%, *p* = 0.1913) following fadraciclib treatment compared with NDC (Fig. 3C).

### Pulsed dosing with fadraciclib in primary AML cells results in apoptosis and reduces cell viability, but is less effective than continuous dosing

To determine if pulsed dosing achieved an optimal response, primary AML cells were treated for up to 8 h with fadraciclib at a concentration of 1 μM. This resulted in a significant increase in %annexinV at both 6 and 8 h (*p* = 0.023 and *p* = 0.0411, respectively) and %active caspase-3 at 6 h (*p* = 0.0351) as compared with NDC (Supplementary Fig. S6A, B). As fadraciclib exerts a rapid effect, we investigated whether a 6-h pulse was as effective as 24-h continuous treatment at inducing apoptosis. Fadraciclib showed a higher %active caspase-3 after continuous treatment compared to the pulsed treatment (Supplementary Fig. S6C). MCL-1 levels were significantly reduced after 24-h continuous treatment compared to both the NDC and the 6-h pulsed treatment (*p* = 0.0167 and *p* = 0.0304, respectively; Supplementary Fig. S6D).

### Increased apoptosis of primary human AML samples in combination studies of fadraciclib + VEN, fadraciclib + AraC, and fadraciclib + AZA

For combination studies, six primary AML samples were selected based on the presence of the most common favorable (e.g., *NPM1*) and unfavorable mutations (e.g., *FLT3*-internal tandem duplication (ITD) and *KMT2A*-PTD), in order to assess the efficacy of fadraciclib and its synergistic activity when combined with VEN, AraC, or AZA, towards these mutations.

Drug combination studies exploit opportunities for reduced drug resistance, decreased toxicity, and efficacy improvement^41^. Based on phase 1 clinical trial data, the maximum clinically achievable concentration of fadraciclib is 6–7 µM when administered as a single agent^42^. In this study, much lower fadraciclib concentrations of 0.25 µM (FAD1) and 0.5 µM (FAD2) in combination with VEN, AraC or AZA were assessed for efficacy against primary AML cells. The concentrations selected were based on previous studies: 0.025 µM (VEN1) and 0.5 µM^43^ (VEN2); 0.01 µM (AraC1) and 0.1 µM^44^ (AraC2); 0.5 µM^45^ (AZA1) and 2 µM^46^ (AZA2), aiming to optimize results for both sensitive and more therapy-resistant patient samples. All drug concentrations were within clinically achievable concentrations (1 µM VEN^47^, 1 µM AraC^48^, and 3–11 µM AZA^49^). Apoptosis, active caspase-3, cell cycle analyses, and proliferation assays were performed at 72 h.

In primary AML samples, %annexinV and %active caspase-3 (Supplementary Figs. S7A–C and S8A–C, respectively) were not significantly different at low concentrations, with any drug combination pair tested. At high concentrations, a modest increase in %annexinV was observed; 82.2 ± 16.9% in FAD2 + VEN2 combination (as compared with 55.7 ± 14% in FAD2, *p* = 0.1059; and 64.6 ± 21.4% in VEN2 alone, *p* = 0.5166) (Supplementary Fig. S7A), 81.6 ± 13.4% in FAD2 + AraC2 combination (as compared with 55.7 ± 14% in FAD2, *p* = 0.1077; and 69.5 ± 22.5% in AraC2 alone, *p* = 0.8117) (Supplementary Fig. S7B), and 87.7 ± 12.3% in FAD2 + AZA2 combination (as compared with 55.7 ± 14% in FAD2, *p* = 0.0109; and 70.7 ± 22.8% in AZA2 alone, *p* = 0.437) (Supplementary Fig. S7C). Consistently, a modest increase in %active caspase-3 was observed at high concentrations; 84.3 ± 11.5% in FAD2 + VEN2 combination (as compared with 55.6 ± 14% in FAD2, *p* = 0.0217; and 65.2 ± 17.9% in VEN2 alone, *p* = 0.267) (Supplementary Fig. S8A), 86.8 ± 5.9% in FAD2 + AraC2 combination (as compared with 55.6 ± 14% in FAD2, *p* = 0.0143; and 69.3 ± 19.5% in AraC2 alone, *p* = 0.4081) (Supplementary Fig. S8B), and 90.7 ± 4% in FAD2 + AZA2 combination (as compared with 55.6 ± 14% in FAD2, *p* = 0.0006; and 74.4 ± 17.3% in AZA2 alone, *p* = 0.3197) (Supplementary Fig. S8C).

Cell cycle analysis revealed %subG0, but no other cell cycle phase, marginally increased in agreement with the increase in %annexinV and %active caspase-3 observed in FAD2 + VEN2, FAD2 + AraC2 and FAD2 + AZA2 combination (Supplementary Fig. S9A–C) as compared with each single drug treatment. Overall results indicate the more complex the molecular genetic lesions or complexity of karyotype, the less efficacious the combination therapy was (Supplementary Table S4).

### Fadraciclib alone and low-dose combination therapies have limited cytotoxicity on normal hematopoietic cells

Fadraciclib alone and in combination with VEN, AraC and AZA was assessed in normal CD34 + hematopoietic samples to determine toxicity. Neither fadraciclib alone nor lower concentration drug combinations exerted a significant increase in %annexinV, %active caspase-3 or %subG0 (Supplementary Figs. S7D–F, S8D–F, and S9D–F, respectively) as compared to NDC. However, as expected, an increase in %annexinV was observed at high concentrations with a corresponding increase in %active caspase-3 (Supplementary Figs. S7D–F and S8D–F, respectively).

### Fadraciclib + AZA combination therapy has a therapeutic window in in vitro cell proliferation assays

**Fig. 4:**
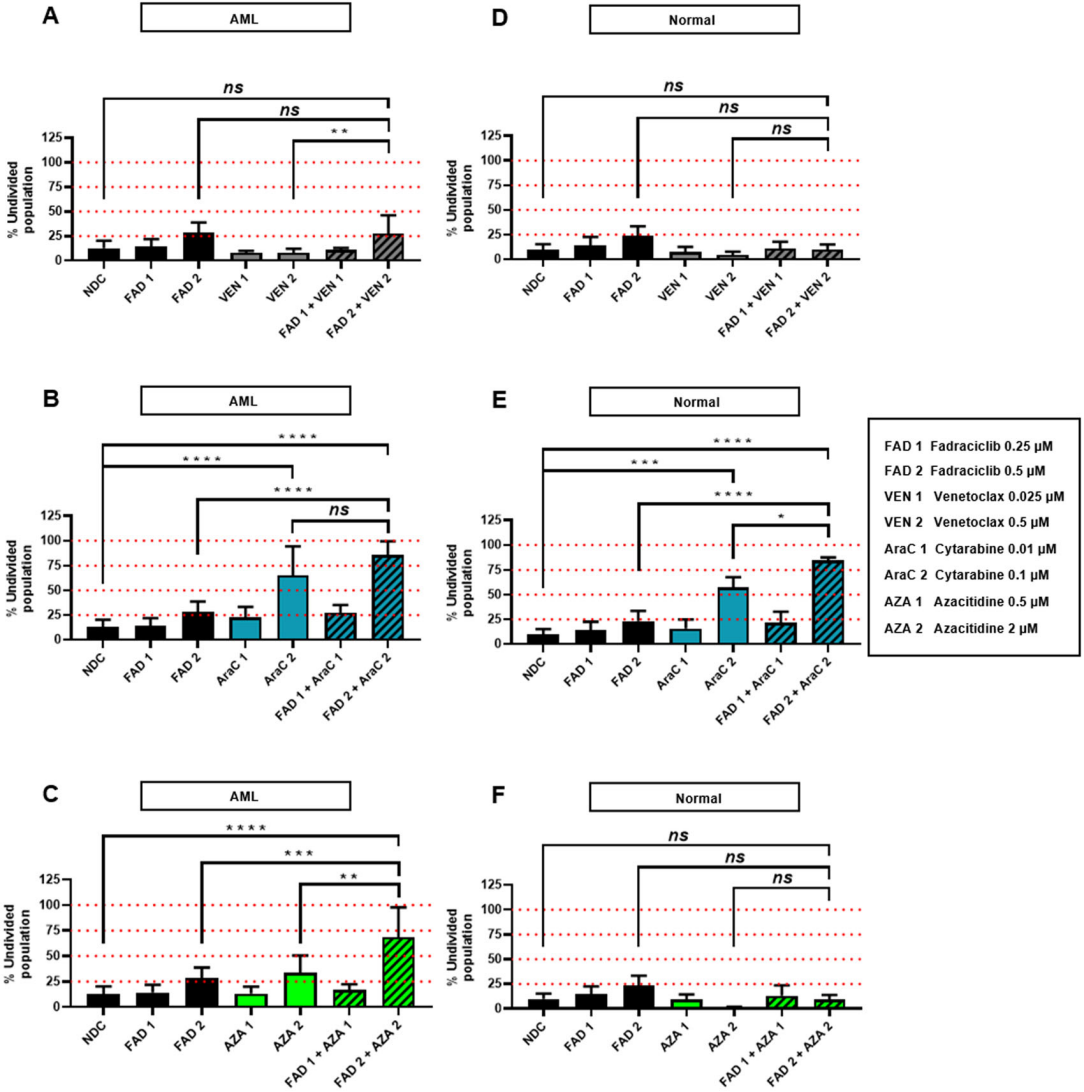
A therapeutic window was observed in cell proliferation assays with the combination of fadraciclib and AZA. **A** Summary bar charts of flow cytometry data of the percentage of undivided populations of primary AML and **D** normal hematopoietic patient samples treated with fadraciclib and/or VEN for 72 h. *n* = 6/3. **B** Summary bar charts of flow cytometry data of the percentage of undivided populations of primary AML and **E** normal hematopoietic patient samples treated with fadraciclib and/or AraC for 72 h. *n* = 6/3. **C** Summary bar charts of flow cytometry data of the percentage of undivided populations of primary AML and **F** normal hematopoietic patient samples treated with fadraciclib and/or AZA for 72 h. *n* = 6/3. Graphs depict mean ± SD (*p < 0.05, **p < 0.01, ***p < 0.001, ****p < 0.0001). Data were compared using ANOVA.

Finally, cell proliferation assays using CellTrace Violet staining were performed. Representative histograms for AML and normal CD34 + samples are shown (Supplementary Fig. S10). When considering the combination therapies, none of the low concentration drug combinations resulted in a significant anti-proliferative effect (increased percentage of undivided population, %Undivided %undivided) compared to either NDC or single drug therapies in either AML or normal CD34 + samples (Fig. 4A–F).

However, differences occurred with the high concentration drug combinations. Specifically, FAD2 + VEN2, had significant anti-proliferative effects when compared to VEN2, but might be explained by the effect of FAD2 (Fig. 4A). This combination had no anti-proliferative effect on normal CD34 + cells (Fig. 4D). Although FAD2 + AraC2, has significantly increased anti-proliferative effect as compared to FAD2 in both AML and normal CD34 + samples, this is fully accounted for by the known cytotoxic effects of AraC (Fig. 4B, E). For FAD2 + AZA2, the anti-proliferative effect in AML samples was significantly increased compared to both FAD2 and AZA2 (%Undivided %undivided of 68.7 ± 29.1% for FAD2 + AZA2 compared with 28.5 ± 10.2% in FAD2, *p* = 0.0004; and 33.5 ± 17% in AZA2, *p* = 0.0024), as well as NDC (Fig. 4C), indicating synergism of this combination with a combination index (CI) of 0.75. No anti-proliferative effect was demonstrated for this combination on normal CD34 + cells (Fig. 4F).

These findings indicate a therapeutic window and provide evidence for further exploration of combination treatment with fadraciclib and AZA as a promising treatment approach.

## Discussion

Our study evaluated the therapeutic potential of fadraciclib in AML. AML cell lines harboring various genetic lesions were tested, these include OCI-AML3 (carries *NPM1* and *DNMT3A*^R882C^ mutations), MOLM-13 (carries *FLT3*-ITD and *KMT2A*-*MLLT3*), and MV4–11 (carries *FLT3*-ITD and *KMT2A*-*AFF1*), aiming to encompass stratification of major risk profiles according to the European LeukemiaNet recommendations^50^. Results indicate MV4–11, harboring adverse prognosis molecular abnormalities, was less sensitive with the highest IC50 (Fig. 1A) and a higher dose of fadraciclib required to induce apoptosis (Fig. 1C–E).

Inhibition of CDK2 and CDK9 was evaluated by phosphorylation of Rb^51,52^ and RNAPII^53,54^. We found the effects of fadraciclib on Rb were cell line specific (Fig. 2A, B), whereas, the endogenous level of RNAPII was decreased in all cell lines (Fig. 2A, C). A transcriptional downregulation of RNAPII is the most plausible explanation for the global gene suppression observed (Fig. 2F and Supplementary Figs. S3–S5). This is consistent with a previous study stating that a downregulation of *POLR2A* encoding the major subunit of RNAPII was observed in leukemic blasts following treatment with alvocidib, which potently inhibits CDK9^55^. A significant decrease in short half-life MCL-1 at gene (Fig. 2G) and protein (Fig. 2A, D) levels highlights a potential target inhibited by fadraciclib.

**Fig. 5:**
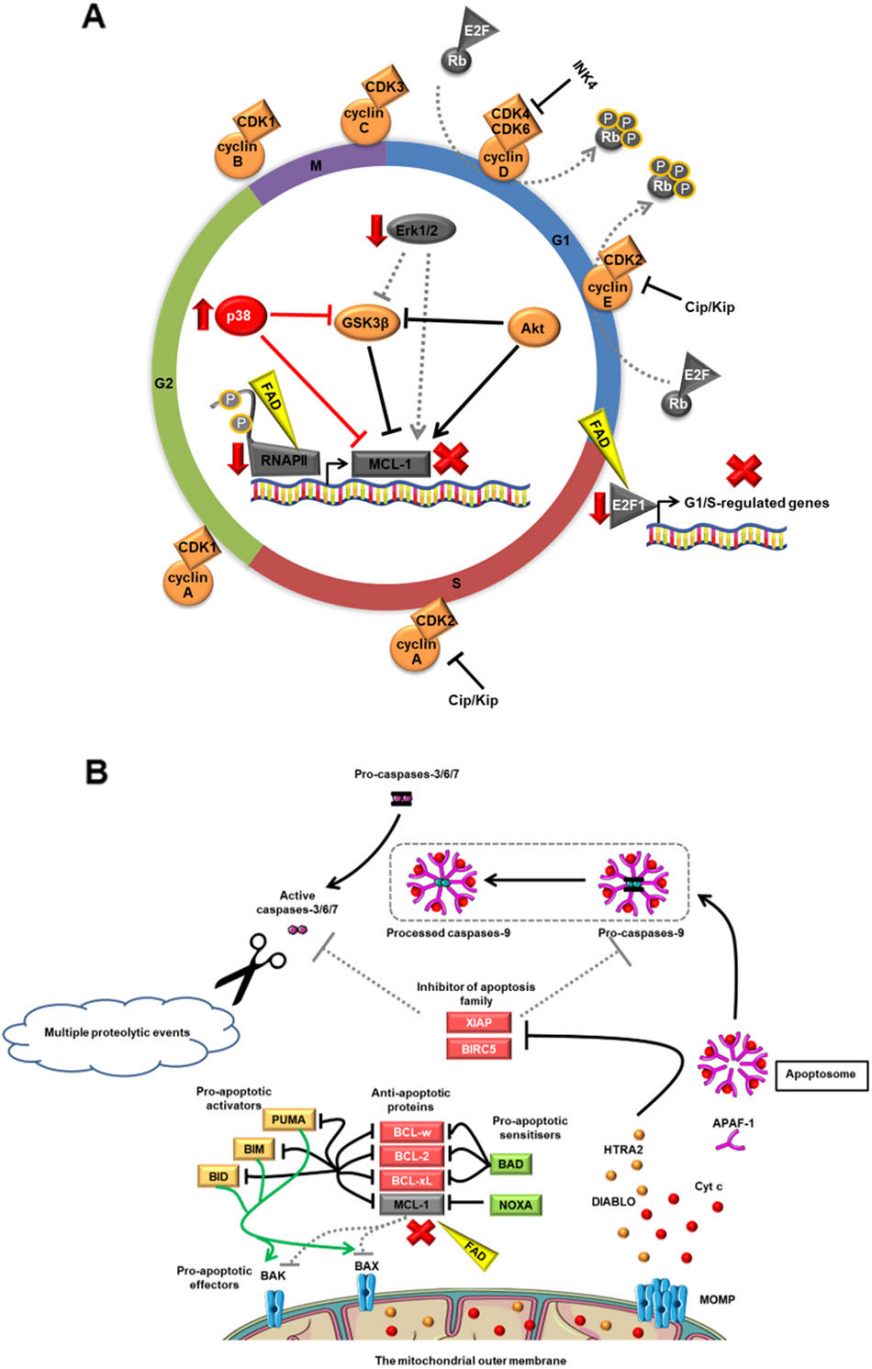
A schematic diagram of the effects of fadraciclib at 4 h. **A** Fadraciclib downregulates *E2F1*, resulting in cell cycle arrest at G1 phase. Downregulation of RNAPII is the most plausible explanation for the global gene suppression observed. A rapid decrease in short half-life MCL-1 level perturbs a balance in the BCL-2 family. A decrease in the Erk1/2 activity observed in all cell lines tested potentially promotes a reduction in MCL-1 stability and half-life. p38 MAPK is markedly increased in MOLM-13 cell line only, implying that the stress response and apoptosis are highly induced. **B** Fadraciclib downregulates MCL-1, which activates the intrinsic apoptosis pathway, followed by cell death. Indeed, a rapid decrease in MCL-1 level perturbs a balance in the BCL-2 family, which leads directly to activator BH3-only proteins-binding BAK and BAX, resulting in their homo-oligomerisation and MOMP. Following this, pro-apoptotic proteins within the mitochondrial intermembrane space, for example, Cyt c, DIABLO and HTRA2 are released. Cyt c binds to APAF-1 to form the apoptosome. Once formed, the apoptosome can then recruit and activate the inactive pro-caspase-9. Following this, caspases-3, 6, and 7 are activated and multiple proteolytic events occur. FAD, Fadraciclib; Cip/Kip, cyclin-dependent kinase interacting protein/kinase inhibitory protein; INK4, inhibitors of cyclin-dependent kinase 4; G1, Gap 1 phase; S, Synthesis phase; G2, Gap 2 phase; M, Mitotic phase; BCL-2, B-cell lymphoma 2; BCL-xL, B-cell lymphoma-extra large; BCL-w, B-cell lymphoma 2-like protein 2; BAD, B-cell lymphoma 2-associated death promoter; NOXA, phorbol-12-myristate-13-acetate-induced protein 1; BIM, B-cell lymphoma 2-like protein 11; BID, BH3 interacting-domain death agonist; PUMA, p53 upregulated modulator of apoptosis; BAK, B-cell lymphoma 2 homologous antagonist/killer; BAX, B-cell lymphoma 2-associated X protein; MOMP, mitochondrial outer membrane permeabilisation; Cyt c, cytochrome c; DIABLO, direct inhibitor of apoptosis proteins-binding protein with low pI; HTRA2, high-temperature requirement A serine peptidase 2; APAF-1, apoptotic protease activating factor-1; BIRC5, baculoviral inhibitor of apoptosis repeat-containing 5; XIAP, X-linked inhibitor of apoptosis protein.

A rapid decrease in MCL-1 level perturbs a balance in the BCL-2 family, activating the intrinsic apoptosis pathway (Fig. 5B). AML cells are more dependent on MCL-1 than normal hematopoietic cells^56^, highlighting the potential of fadraciclib as a therapeutic approach for AML. Downregulation of *E2F1* (Fig. 2G) may explain the G1 cell cycle arrest seen in OCI-AML3 and MV4–11 following fadraciclib treatment (Supplementary Fig. S1). Interestingly, Erk1/2 were suppressed in all cell lines tested (Supplementary Fig. S2A, D), which potentially promotes a reduction in MCL-1 stability and half-life^57^. In various cancer cell lines, siRNA-mediated *PPP1R10* knockdown results in tumor suppressor PTEN release from nuclear sequestration^58^. Hence, the significant decrease in *PPP1R10* gene expression observed (Fig. 2G) potentially promotes an induction of apoptosis. Using leukemic blasts from adult patients with refractory AML in a phase 1 clinical trial (NCT00470197), treatment with the pan-CDKi alvocidib, resulted in a downregulation of various genes^55^. Among these, downregulation of *POLR2A*, encoding the major subunit of RNAPII, and *E2F1* was seen. In addition, a decrease in phosphorylation of RNAPII and expression of MCL-1 was observed in leukemia blasts of some patients treated with alvocidib who achieved a CR^59^. Favorably, we found that the function of CDK1, which plays an important role in mitosis^60,61^, was not perturbed, suggesting better immune function with less impact on normal hematopoietic cells following fadraciclib treatment.

Recently, a phase 1 clinical trial of fadraciclib in solid tumor patients was completed (NCT02552953)^42^. Dose-limiting toxicities were reversible and a biologically effective dose of 192 mg/m^2^/day, which corresponds to the drug concentration of 6–7 μM in vitro was established^42^.

Over the past few years, many targeted therapies for patients with AML have emerged with efficacy dependent on molecular subtype of AML^62^. This highlights the important of assessing the efficacy of fadraciclib on specific AML subtypes based on its mechanism of action of inhibiting CDK9. As part of the Super Elongation Complex (SEC), CDK9 is important for transcriptional elongation, in particular in *KMT2A*-r AML^63^, it is therefore rational to evaluate the efficacy of fadraciclib in this AML subtype. Preliminary results indicate *KMT2A*-r AML cell lines are more sensitive to fadraciclib^29^. Patient samples, however, display a high diversity of genetic mutations (Table. 1). We therefore observed a more variable fadraciclib response with *KMT2A*-PTD status not being a predictor for better treatment response. This is consistent with previous results showing that the CDK9-specific inhibitor atuveciclib inhibits the proliferation of seven AML cell lines, regardless of *KMT2A*-r status^64^. In addition, atuveciclib displayed potent in vitro activity in 80% of AML patient samples harboring *KMT2A* WT, including those with mutant *NPM1* or *FLT3*-ITD.

Considering the combination studies, primary AML samples were selected based on the most common favorable (e.g., *NPM1*) and unfavorable mutations (e.g., *FLT3*-ITD and *KMT2A*-PTD). Initially, we hypothesized that the treatment response would be relevant to the risk profiles of the individual genetic lesions in primary AML samples. However, we found that complexity of the molecular genetic lesions and karyotypes have more influence. The more complex the molecular landscape, the less efficacious the combination therapy (Supplementary Table S4). The treatment response variability associated with the high diversity of genetic mutations observed reiterates the results of previous experiment showing the variable fadraciclib response, regardless of *KMT2A*-PTD status. Taken together, our results highlight the importance of genetic and molecular profiling prior to initiating therapy, to identify personalized therapeutic combinations most likely to benefit individual patient. Regarding the 2017 ELN risk stratification^50^, next generation sequencing (NGS), exome sequencing and genome wide assays^65^, are being developed to replace single gene assays. This will facilitate better prognostication and more personalized therapy in the future^66^. A short delay in commencing therapy in newly diagnosed AML had no effect on outcome after accounting for other prognostic covariates^67^, providing a potential window to perform NGS.

As a global effect, an increase in cell death in primary human AML cells was shown for apoptosis, active caspase-3 assays, and cell cycle analysis when fadraciclib was combined with VEN, AraC, or AZA. In primary AML cells, combining the MCL-1 inhibitor S63845 with VEN results in greater efficacy than either inhibitor alone, with a more potent activity against leukemic rather than normal hematopoietic progenitors^68^. In an AML patient-derived xenograft mouse model bearing *FLT3*-ITD, the CDK9 inhibitor A-1592668 combined with VEN provided a significant survival advantage over single treatments^69^. In a lymph node mimicking microenvironment, fadraciclib in combination with VEN for 24 h efficiently induced apoptosis of primary CLL cells^70^, a disease where MCL-1 plays a role in disease progression and fludarabine resistance^71^. Ongoing phase 1 clinical trials are evaluating the safety and tolerability of fadraciclib in combination with VEN in patients with relapsed/refractory AML or myelodysplastic syndromes (MDS) (NCT04017546); and relapsed/refractory CLL (NCT03739554).

When considering combining CDK9 inhibitors with AraC, a recent phase 2 study (NCT01349972), demonstrated a significantly higher efficacy of FLAM (alvocidib, cytarabine plus mitoxantrone) as compared with the standard “7 + 3” regimen in terms of a CR rate^26,28^. However, no increase in overall or event-free survival rates were observed. Potentially, this may be due to the higher toxicity of the combination regimen based on our preliminary data showing the impact of the fadraciclib + AraC on normal hematopoietic cells.

In preliminary experiments, pre-treatment with alvocidib reduced the IC50 of AZA in MV4–11 cell line^72^. In the same manner seen in fadraciclib + AZA combination presented here, alvocidib + AZA also increased %active caspase-3 as compared with single treatments. In a MOLM-13 xenograft model, the combination showed greater tumor growth inhibition compared to single agent treatments. Our results, demonstrated a potential therapeutic window with the FAD2 + AZA2 combination, as measured by %Undivided %undivided of AML cells in cell proliferation assay (Fig. 4C). These interesting results identify an attractive therapeutic strategy warranting consideration of clinical development in AML.

In summary, our studies indicate the preclinical efficacy of the CDK2/9 inhibitor fadraciclib as a single agent or in conjunction with frontline AML chemotherapeutics, highlighting its potential as a novel therapeutic approach. Our work implicates the importance of molecular genetic profiling as a critical step prior to initiating therapy for AML, highlighting the need for a more personalized medicine approach to improve outcomes for patients.

## Materials and methods

### Cell lines and primary AML samples cell culture

OCI-AML3, MOLM-13, and MV4–11 (DSMZ) were cultured at 37 °C in a 5% CO_2_ atmosphere in RPMI 1640 media (Sigma-Aldrich) with 20% or 10% fetal bovine serum (Gibco), supplemented with 1 mM glutamine and 1% penicillin-streptomycin (Invitrogen).

Healthy donor and diagnostic primary AML samples were taken in accordance with the Declaration of Helsinki, and Ethics Committee approval (15/WS/0077). Demographic data, cytogenetic abnormalities and genetic lesions of AML patients are shown in Table. 1. Primary samples were thawed and cultured overnight in serum-free medium II (Stem Cell Technologies) supplemented with hIL-3, hIL-6, hSCF, and hFLT3L (all at 10 ng/mL) (Stem Cell Technologies).

### Inhibitors

Ten micromolar stock solutions of fadraciclib (Cyclacel), AZA (Stratech), and VEN (Stratech) were prepared in DMSO. 10 mM AraC (Sigma-Aldrich) was prepared in water. All stocks were stored at −20 °C and dilutions freshly prepared in cell culture media.

### Rezasurin reduction assay

Half-maximal inhibitory concentration (IC50) of AML cell lines treated with fadraciclib for up to 72 h was established using 7-Hydroxy-3H-phenoxazin-3-one-10-oxide sodium salt resazurin assay (Sigma-Aldrich) according to manufacturer’s instructions. IC50 were calculated using GraphPad Prism 8 software (GraphPad Software).

### Gene expression analysis

RNA was extracted using RNA Easy Micro kits (Qiagen) and converted to cDNA using high-capacity cDNA reverse transcription kit (Applied Biosystems). Quantitative reverse transcription polymerase chain reaction (qRT-PCR) was performed using Fluidigm Biomark technology and data were collected as per manufacturer’s instructions. Data was analyzed using the 2^−ΔΔCT^ compared to no drug control (NDC) as previously described^73^. Internal sample control was ensured by subtracting the average of six housekeeping genes *ATP5F1B*, *B2M*, *CYC1*, *RNF20*, *TYW1*, and *UBE2D2* from the Ct value of each gene of interest. Mean and standard deviation of fold change in expression were calculated. For Primer sequences see Supplementary Table S1.

### Protein extraction and quantification

In all, 1–5 × 10^6^ cells were harvested and washed twice in ice-cold PBS and pelleted at 300 × *g* for 5 min. Then, the pellets were lyzed in ice-cold solubilization buffer containing phosphatase and protease inhibitors at a concentration of 1 × 10^6^ cells per 10 μl solubilization buffer (50 mM Tris-HCl pH 7.5, 150 mM NaCl, 1% Nonidet P40, 10% Glycerol, cOmplet ULTRA 1 tablet and PhosSTOP phosphatase inhibitor 1 tablet per 10 mL of solubilization buffer). This suspension was left on ice and vortexed every 15 min for 1 h, then centrifuged at 14,000 × *g* for 10 min to pellet all cell debris. The supernatant was transferred to a fresh eppendorf and stored at −80 ^o^ C until required.

Protein concentration was quantified using the Quick Start Bradford Dye reagent and compared to a standard curve prepared using Quick StartTM BSA protein. The colorimetric protein assay was measured by visual absorbance on the Spectramax M5 plate reader at 595 nm at using SoftMax Pro software.

### Protein analysis

Cell lysates were prepared and 30 µg of protein was resolved. Western blotting was performed using the NuPAGE electrophoresis system (Invitrogen) as per manufacturer’s instructions, and protein detection via Odyssey Fc Imaging System. Densitometry was performed using Image Studio Lite version 5.2. Antibodies and experimental conditions are outlined in Supplementary Table S2.

### Flow cytometry

For proliferation analysis, primary AML and normal CD34+ cells were stained with CellTrace Violet (Thermo Fisher Scientific) as per manufacturer’s protocol. For establishing a maximum point of fluorescence staining, cells were cultured with Colcemid (100 ng/mL, Sigma-Aldrich) to determine non-dividing cells. All primary cells were treated with fadraciclib, VEN, AraC, AZA or fadraciclib in combination with VEN, AraC or AZA in physiological growth factor conditions. Annexin V/7-aminoactinomycin D (7-AAD) or DAPI (4’,6-diamidino-2-phenylindole) (BD Biosciences) staining to assess apoptosis by flow cytometry using 1 × 10^5^ cells per condition. Propidium iodide (PI) staining buffer to assess cell cycle progression as per manufacturer’s protocol. Fixation/Permeabilization Solution Kit (BD Biosciences) was used prior to MCL-1 or active caspase-3 staining. For staining reagents used, see Supplementary Table S3.

### Combination studies

CompuSyn software (ComboSyn, Inc) was used to investigate the synergism in the cell proliferation assays. The software was based on the Chou-Talalay method for drug combination based on the median-effect equation derived from the mass-action law principle^74^. The CI provides a quantitative definition for additive effect (CI = 1), synergism (CI < 1) or antagonism (CI > 1) in drug combinations.

### Statistics

Average responses from at least three independent experiments are shown (mean ± SD). Statistical analysis used GraphPad Prism 8 for Student’s *t*-test and one-way ANOVA (*p < 0.05, **p < 0.01, ***p < 0.001, ****p < 0.0001).

## Supplementary information

Supplementary Figure Legends

Supplementary Table S1

Supplementary Table S2

Supplementary Table S3

Supplementary Table S4

Supplementary Fig. S1

Supplementary Fig. S2

Supplementary Fig. S3

Supplementary Fig. S4

Supplementary Fig. S5

Supplementary Fig. S6

Supplementary Fig. S7

Supplementary Fig. S8

Supplementary Fig. S9

Supplementary Fig. S10
