# Correction to: Interrogation of novel CDK2/9 inhibitor fadraciclib (CYC065) as a potential therapeutic approach for AML

**DOI:** 10.1038/s41420-021-00558-1

**Published:** 2021-07-06

**Authors:** Wittawat Chantkran, Ya-Ching Hsieh, Daniella Zheleva, Sheelagh Frame, Helen Wheadon, Mhairi Copland

**Affiliations:** 1 Paul O’Gorman Leukaemia Research Centre, Institute of Cancer Sciences, College of Medical, Veterinary and Life Sciences, University of Glasgow, Glasgow, UK; 2 Department of Pathology, Phramongkutklao College of Medicine, Bangkok, Thailand; 3 Cyclacel Limited, Dundee, UK

**Keywords:** Acute myeloid leukaemia, Preclinical research

Correction to: *Cell Death Discovery* **7**:137; 10.1038/s41420-021-00496-y; published online 10 June 2021

The original version of this article unfortunately contained a mistake in the funding section. This research was funded by a Ph.D. studentship from the Royal Thai Government. Additional funding was provided by Cyclacel Ltd. This study was supported by the Glasgow Experimental Cancer Medicine Center, which is funded by Cancer Research UK [C58789/A251741] and the Chief Scientist’s Office, Scotland. Cell-sorting facilities were funded by the Kay Kendall Leukemia Fund (KKL501) and the Howat Foundation. The authors apologize for the mistake. The original article has been corrected.

