# Supplementary material for: Interrogation of novel CDK2/9 inhibitor fadraciclib (CYC065) as a potential therapeutic approach for AML
Source: Cell Death Discov. 2021 Jun 10;7:137. doi: 10.1038/s41420-021-00496-y (PMC8192769; doi:10.1038/s41420-021-00496-y)
Supplement: Supplementary file 1 — Supplementary Figure Legends [file 41420_2021_496_MOESM1_ESM.docx]

**Supplementary Fig. S1 Cell cycle arrest at G1 phase was observed in OCI-AML3 and MV4-11, but not MOLM-13, cells.** Flow cytometric analysis of cell cycle progression using PI staining in OCI-AML3, MOLM-13, MV4-11 cells treated with fadraciclib for 4 h. *n* = 3. Graphs depict mean ± SD (**p* < 0.05; ***p* < 0.01; ****p* < 0.001; *****p* < 0.0001). Data were compared using ANOVA.

**Supplementary Fig. S2 The effects of fadraciclib on target protein expression. A** Western blotting of OCI-AML3, MOLM-13, MV4-11 cell lines treated with 1 µM of fadraciclib for 4 and 24 h. SH-PTP2 was used as an internal protein loading control. **B** Densitometric analysis of threonine 320 (T320) phosphorylated and total protein phosphatase 1 alpha (PP1α). **C** Densitometric analysis of threonine180/tyrosine 182 (T180/Y182) phosphorylated and total p38 MAPK. **D** Densitometric analysis of threonine 202/tyrosine 204 (T202/Y204) phosphorylated and total extracellular signal-regulated protein kinases 1 and 2 (ERK1/2). **E** Densitometric analysis of serine 473 (S473) phosphorylated and total Akt. **F** Densitometric analysis of serine 9 (S9) phosphorylated and total glycogen synthase kinase 3 beta (GSK3β). *n* = 3. Graphs depict mean ± SD (**p* < 0.05; ***p* < 0.01; ****p* < 0.001; *****p* < 0.0001). Data were compared using the Student’s *t*-test.

**Supplementary Fig. S3 The effects of fadraciclib on gene expression of cyclin-dependent kinases, transcription factors and protein phosphatases**. OCI-AML3, MOLM-13 and MV4-11 cell lines were treated with 0.75 µM, 0.5 µM and 1 µM of fadraciclib, respectively, for 4 and 24 h. **A** Fold change expression of CDK genes relative to NDC. **B** Fold change expression of transcription factor and key protein phosphatase genes relative to NDC. *n* = 3. Graphs depict mean ± SD (**p* < 0.05; ***p* < 0.01; ****p* < 0.001; *****p* < 0.0001). Data were compared using the Student’s t-test.

**Supplementary Fig. S4 The effects of fadraciclib on gene expression of cyclin-dependent kinase inhibitors and DNA damage response regulators.** OCI-AML3, MOLM-13 and MV4-11 cell lines were treated with 0.75 µM, 0.5 µM and 1 µM of fadraciclib, respectively, for 4 and 24 h. **A** Fold change expression of CDK inhibitor genes relative to NDC. **B** Fold change expression of DNA damage response regulator genes relative to NDC. *n* = 3. Graphs depict mean ± SD (**p* < 0.05; ***p* < 0.01; ****p* < 0.001; *****p* < 0.0001). Data were compared using the Student’s t-test.

**Supplementary Fig. S5 The effects of fadraciclib on gene expression of anti-and pro-apoptotic related genes.** OCI-AML3, MOLM-13 and MV4-11 cell lines were treated with 0.75 µM, 0.5 µM and 1 µM of fadraciclib, respectively, for 4 and 24 h. **A** Fold change expression of anti-apoptotic related genes relative to NDC. **B** Fold change expression of pro-apoptotic related genes relative to NDC. *n* = 3. Graphs depict mean ± SD (**p* < 0.05; ***p* < 0.01; ****p* < 0.001). Data were compared using the Student’s t-test.

**Supplementary Fig. S6 Pulsed treatment with fadraciclib in primary AML cells resulted in apoptosis and reduced cell viability, but was less effective than 24 h-continuous treatment. A** Representative flow cytometry plots and summary bar charts of the percentage of annexin V-positive cells of primary AML scells following treatment with 0.5 µM or 1 µM of fadraciclib for 2-8 h. **B** Representative flow cytometry plots and summary summary bar charts of the percentage of active caspase-3-positive cells of primary AML cells following treatment with 0.5 µM or 1 µM of fadraciclib for 2-8 h. **C** Representative flow cytometry plots and summary bar charts of the percentage of active caspase-3-positive cells of primary AML cells following 6-h pulsed fadraciclib treatment or 24-h continuous fadraciclib treatment. **D** Representative flow cytometry plots and summary bar charts of the percentage of MCL-1 positive cells in primary AML cells following 6-h pulsed fadraciclib treatment or 24-h continuous fadraciclib treatment. Graphs depict means ± SD (**p* < 0.05). Data were compared using ANOVA.

**Supplementary Fig. S7 An increase in the percentage of annexin V-positive cells of primary AML samples was observed in the combination studies of fadraciclib+VEN, fadraciclib+AraC, and fadraciclib+AZA. A** Summary bar charts of flow cytometry data of the percentage of annexin V-positive cells of primary AML and **D** normal hematopoietic patient samples treated with fadraciclib and/or VEN for 72 h. *n* = 6/3. **B** Summary bar charts of flow cytometry data of the percentage of annexin V-positive cells of primary AML and **E** normal hematopoietic patient samples treated with fadraciclib and/or AraC for 72 h. *n* = 6/3. **C** Summary bar charts of flow cytometry data of the percentage of annexin V-positive cells of primary AML and **F** normal hematopoietic patient samples treated with fadraciclib and/or AZA for 72 h. *n* = 6/3. Graphs depict mean ± SD (**p* < 0.05; ***p* < 0.01; ****p* < 0.001; *****p* < 0.0001). Data were compared using ANOVA.

**Supplementary Fig. S8 An increase in the percentage of active caspase-3-positive cells of primary AML samples was observed in the combination studies of fadraciclib+VEN, fadraciclib+AraC, and fadraciclib+AZA. A** Summary bar charts of flow cytometry data of the percentage of active caspase-3-positive cells of primary AML and **D** normal hematopoietic patient samples treated with fadraciclib and/or VEN for 72 h. *n* = 6/3. **B** Summary bar charts of flow cytometry data of the percentage of active caspase-3-positive cells of primary AML and **E** normal hematopoietic patient samples treated with fadraciclib and/or AraC for 72 h. *n* = 6/3. **C** Summary bar charts of flow cytometry data of the percentage of active caspase-3-positive cells of primary AML and **F** normal hematopoietic patient samples treated with fadraciclib and/or AZA for 72 h. *n* = 6/3. Graphs depict mean ± SD (**p* < 0.05; ***p* < 0.01; ****p* < 0.001; *****p* < 0.0001). Data were compared using ANOVA.

**Supplementary Fig. S9 An increase in the percentage of sub G0 population of primary AML samples was observed in the combination studies of fadraciclib+VEN, fadraciclib+AraC, and fadraciclib+AZA. A** Summary bar charts of flow cytometry data of cell cycle phase of primary AML and **D** normal hematopoietic patient samples treated with fadraciclib and/or VEN for 72 h. *n* = 6/3. **B** Summary bar charts of flow cytometry data of cell cycle phase of primary AML and **E** normal hematopoietic patient samples treated with fadraciclib and/or AraC for 72 h. *n* = 6/3. **C** Summary bar charts of flow cytometry data of cell cycle phase of primary AML and **F** normal hematopoietic patient samples treated with fadraciclib and/or AZA for 72 h. *n* = 6/3. Graphs depict mean ± SD (**p* < 0.05; ***p* < 0.01; ****p* < 0.001; *****p* < 0.0001). Data were compared using ANOVA.

**Supplementary Fig. S10** **Representative flow cytometry histograms of cell proliferation assays. A** Representative histograms of cell proliferation assays of primary AML sample (AML 44). **B** Representative histograms of cell proliferation assays of normal CD34+ hematopoietic patient sample.
