# Supplementary material for: Interrogation of novel CDK2/9 inhibitor fadraciclib (CYC065) as a potential therapeutic approach for AML
Source: Cell Death Discov. 2021 Jun 10;7:137. doi: 10.1038/s41420-021-00496-y (PMC8192769; doi:10.1038/s41420-021-00496-y)
Supplement: Supplementary file 2 — Supplementary Table S1 [file 41420_2021_496_MOESM2_ESM.docx]

**Supplementary Table S1. List of primers used in this study**

| **Primer** | **Sequence 5´ to 3´** | **Accession** |
| --- | --- | --- |
| *ATM* Forward | CGG AGC TGA TTG TAG CAA CAT ACT A | NM_001351834.2 |
| *ATM* Reverse | CAG ATA GAG CCT GAA GTA CAC AGA G |  |
| *ATR* Forward | CAG CTC TCT ATG AAG GCC ATT CAA | NM_001184.4 |
| *ATR* Reverse | GTT CTA CTG TTT CAC TGT CTG TTG C |  |
| *BCL2* Forward | CCC TGT GGA TGA CTG AGT ACC | NM_000633.3 |
| *BCL2* Reverse | GTT CCA CAA AGG CAT CCC AGC |  |
| *BAK1* Forward | TCA TCG GGG ACG ACA TCA AC | NM_001188.4 |
| *BAK1* Reverse | CAA ACA GGC TGG TGG CAA TC |  |
| *BAX* Forward | GAC ATT GGA CTT CCT CCG GG | NM_001291428.2 |
| *BAX* Reverse | ACA GGG ACA TCA GTC GCT TC |  |
| *BIRC5* Forward | CCA GAT GAC GAC CCC ATA GAG G | NM_001168.3 |
| *BIRC5* Reverse | TGG CTC TTT CTC TGT CCA GTT TC |  |
| *CDC25A* Forward | GTC TAG ATT CTC CTG GGC CAT TG | NM_001789.3 |
| *CDC25A* Reverse | CAG AAT GGC TCC TCT TCA GAG C |  |
| *CDC25B* Forward | GGA TTT GTG GAC ATC CTA GAG AGT | NM_021873.3 |
| *CDC25B* Reverse | ACT TGC TGT ACA TGA CGA GGT |  |
| *CDC25C* Forward | CAC TCA GCT TAC CAC TTC TGC AG | NM_001790.5 |
| *CDC25C* Reverse | GGG CTA CAT TTC ATT AGG TGC TGG |  |
| *CDK1* Forward | ATG AAG TGT GGC CAG AAG TG | NM_001786.5 |
| *CDK1* Reverse | CAG AAA TTC GTT TGG CTG GAT CA |  |
| *CDK2* Forward | GCT TGT TAT CGC AAA TGC TGC | NM_001290230.2 |
| *CDK2* Reverse | GAT GGG GTA CTG GCT TGG TC |  |
| *CDK3* Forward | TGG TGA CAC TGT GGT ATC GC | NM_001258.2 |
| *CDK3* Reverse | GGG CTT TTC GAG TCA CCA TC |  |
| *CDK4* Forward | CCC ATC AGC ACA GTT CGT GA | NM_000075.4 |
| *CDK4* Reverse | AAC ACC AGG GTT ACC TTG ATC TC |  |
| *CDK5* Forward | TCT TCC AGC TAC TAA AAG GGC TG | NM_004935.4 |
| *CDK5* Reverse | CAA TTT CAG CTC CCC ATT CCT G |  |
| *CDK6* Forward | CCG AAG TCT TGC TCC AGT CC | NM_001259.8 |
| *CDK6* Reverse | GTT GAT CAA CAT CTG AAC TTC CAC G |  |
| *CDK7* Forward | GTG GCC GGA CAT GTG TAG TC | NM_001324070.2 |
| *CDK7* Reverse | GCC GTA ATT CGA GCA CAT GG |  |
| *CDK9* Forward | ATG GAA AAC GAG AAG GAG GGG | NM_001261.4 |
| *CDK9* Reverse | TAG GGG GAA GCT TTG GTT CG |  |
| *CDKN1A* Forward | ACA GCA GAG GAA GAC CAT GTG | NM_000389.5 |
| *CDKN1A* Reverse | GGA GTG GTA GAA ATC TGT CAT GC |  |
| *CDKN2C* Forward | CGT CAA TGC ACA AAA TGG ATT TGG | NM_078626.3 |
| *CDKN2C* Reverse | GAA TGA CAG CGA AAC CAG TTC GG |  |
| *CDKN2D* Forward | GTG CAT CCC GAC GCC CTC AAC | NM_001800.4 |
| *CDKN2D* Reverse | TGG CAC CTT GCT TCA GCA GCT C |  |
| *CDKN3* Forward | GGT TTA TGT GCT CTT CCA GGT TG | NM_005192.4 |
| *CDKN3* Reverse | GTG CAG CTA ATT TGT CCC GAA AC |  |
| *CHEK1* Forward | GGT CAC AGG AGA GAA GGC AAT A | NM_001114122.2 |
| *CHEK1* Reverse | GGA AGA ATC TCT GAG CAT CTG G |  |
| *CHEK2* Forward | AGT GGA TCC AAA GGC ACG TT | NM_007194.4 |
| *CHEK2* Reverse | CCT GGG GTA GAG CTG TGG AT |  |
| *E2F1* Forward | GAT CAA AGC CCC TCC TGA GAC | NM_005225.3 |
| *E2F1* Reverse | ATC CCA CCT ACG GTC TCC TC |  |
| *E2F2* Forward | GGC TGG CCT ATG TGA CTT ACC | NM_004091.4 |
| *E2F2* Reverse | GGT TGT CCT CAG TCC TGT CG |  |
| *E2F4* Forward | GCT GAC ACC CTA GCT GTA CG | NM_001950.4 |
| *E2F4* Reverse | AAT CTC CCG GGT ATT GCA GC |  |
| *MCL1* Forward | GCC TTC CAA GGA TGG GTT TG | NM_182763.2 |
| *MCL1* Reverse | TAT GCC AAA CCA GCT CCT ACT C |  |
| *MYC* Forward | CGT CCT CGG ATT CTC TGC TC | NM_002467.6 |
| *MYC* Reverse | CTT GTT CCT CCT CAG AGT CGC |  |
| *PPP1R10* Forward | TCC CCC TAG ATG AGG AGT GTT C | NM_002714.4 |
| *PPP1R10* Reverse | AGA ACT GGA GGC AAC TTG GAG |  |
| *TP53* Forward | GAG CTG AAT GAG GCC TTG GA | NM_000546.6 |
| *TP53* Reverse | CTG AGT CAG GCC CTT CTG TCT T |  |
| *XIAP* Forward | AGG GCT AAC TGA TTG GAA GCC | NM_001204401.2 |
| *XIAP* Reverse | GTT CTT ACC AGA CAC TCC TCA AG |  |
| **Housekeeping genes** | | |
| *ATP5F1B* Forward | GCT GAG CTG GGC ATC TAT CC | NM_001686.4 |
| *ATP5F1B* Reverse | TGG AGG GAT TTG TAG TCC TGC |  |
| *B2M* Forward | TTG TCT TTC AGC AAG GAC TGG | NM_004048.4 |
| *B2M* Reverse | ATG CGG CAT CTT CAA ACC TCC |  |
| *CYC1* Forward | ACT GCG GGA AGG TCT CTA CTT | NM_001916.5 |
| *CYC1* Reverse | GGG TGC CAT CGT CAA ACT CTA |  |
| *RNF20* Forward | GGT GTC TCT TCA ACG GAG GAA | NM_019592.7 |
| *RNF20* Reverse | TAG TGA GGC ATC ATC AGT GGC |  |
| *TYW1* Forward | ATT GTC ATC AAG ACG CAG GGC | NM_018264.4 |
| *TYW1* Reverse | GTT GCG AAT CCC TTC GCT GTT |  |
| *UBE2D2* Forward | CCA TGG CTC TGA AGA GAA TCC | NM_003339.3 |
| *UBE2D2* Reverse | GAT AGG GAC TGT CAT TTG GCC |  |
