# Supplementary material for: Interrogation of novel CDK2/9 inhibitor fadraciclib (CYC065) as a potential therapeutic approach for AML
Source: Cell Death Discov. 2021 Jun 10;7:137. doi: 10.1038/s41420-021-00496-y (PMC8192769; doi:10.1038/s41420-021-00496-y)
Supplement: Supplementary file 3 — Supplementary Table S2 [file 41420_2021_496_MOESM3_ESM.docx]

**Supplementary Table S2. List of antibodies used and the experimental conditions for Western blotting**

| **Primary antibody** | **Species** | **Dilution** | **Diluent** | **Source** | **Catalog number** |
| --- | --- | --- | --- | --- | --- |
| Akt (phospho S473) (D9E) XP | Rabbit | 1:1000 | 3% BSA | Cell Signalling Technology, London, UK | 4060 |
| Akt (total) | Mouse | 1:1000 | 5% milk | Santa Cruz Biotechnology, Heidelberg, Germany | sc-5298 |
| Erk1/2 (phospho T202/Y204) | Rabbit | 1:1000 | 3% BSA | Chemicon-Millipore, Feltham, UK | AB3826 |
| Erk1/2 (total) | Mouse | 1:1000 | 5% milk | Santa Cruz Biotechnology, Heidelberg, Germany | sc-514302 |
| GSK3β (phospho S9) | Rabbit | 1:1000 | 3% BSA | Cell Signalling Technology, London, UK | 9336 |
| GSK3β (total) | Mouse | 1:1000 | 5% milk | Santa Cruz Biotechnology, Heidelberg, Germany | sc-377213 |
| MCL-1 | Rabbit | 1:500 | 5% milk | Cell Signalling Technology, London, UK | 4572S |
| p38 MAPK (phospho T180/Y182) | Rabbit | 1:500 | 3% BSA | Cell Signalling Technology, London, UK | 9211 |
| p38 MAPK (total) | Rabbit | 1:1000 | 5% milk | Cell Signalling Technology, London, UK | 9212 |
| PARP1 | Rabbit | 1:1000 | 5% milk | Cell Signalling Technology, London, UK | 9542 |
| PP1α (phospho T320) | Rabbit | 1:1000 | 3% BSA | Cell Signalling Technology, London, UK | 2581 |
| PP1α Antibody (total) | Rabbit | 1:1000 | 5% milk | Cell Signalling Technology, London, UK | 2582 |
| Rb (phospho S807/S811) | Rabbit | 1:1000 | 3% BSA | Cell Signalling Technology, London, UK | 9308 |
| Rb (4H1) Mouse mAb (total) | Mouse | 1:1000 | 5% milk | Cell Signalling Technology, London, UK | 9309 |
| RNAPII (phospho S2) | Rabbit | 1:1000 | 3% BSA | Abcam, Cambridge, UK | ab70324 |
| RNAPII RPB1 CTD (4H8) (total) | Mouse | 1:1000 | 5% milk | Cell Signalling Technology, London, UK | 2629 |
| SH-PTP2 | Mouse | 1:5000 | 5% milk | Santa Cruz Biotechnology, Heidelberg, Germany | sc-7384 |
| **Secondary antibody** |  |  |  |  |  |
| IRDye 680RD Donkey anti-Mouse IgG | Mouse | 1:5000 | 5% milk | LI-COR Biosciences, Cambridge, UK | 925-68072 |
| IRDye 800CW Donkey anti-Rabbit IgG | Rabbit | 1:5000 | 5% milk | LI-COR Biosciences, Cambridge, UK | 925-32213 |
