# Supplementary material for: Interrogation of novel CDK2/9 inhibitor fadraciclib (CYC065) as a potential therapeutic approach for AML
Source: Cell Death Discov. 2021 Jun 10;7:137. doi: 10.1038/s41420-021-00496-y (PMC8192769; doi:10.1038/s41420-021-00496-y)
Supplement: Supplementary file 4 — Supplementary Table S3 [file 41420_2021_496_MOESM4_ESM.docx]

**Supplementary Table S3. List of staining reagents used in flow cytometric analysis**

| **Primary antibody** | **Fluorochrome** | **Source** | **Catalog number** |
| --- | --- | --- | --- |
| 7-AAD | - | BD Biosciences, Wokingham, UK | 559925 |
| Active caspase-3 (Rabbit) | PE | BD Biosciences, Wokingham, UK | 550821 |
| Annexin V | FITC | BD Biosciences, Wokingham, UK | 556419 |
| CellTrace Violet proliferation kit | - | Thermo Fisher Scientific, Loughborough, UK | C34557 |
| DAPI | - | BD Biosciences, Wokingham, UK | 564907 |
| MCL-1 (D2W9E) | Alexa Fluor 647 | Cell Signalling Technology, London, UK | 78471 |
| PI/RNase staining buffer | - | BD Biosciences, Wokingham, UK | 550825 |
| Rabbit (DA1E) mAb IgG XP Isotype Control (for active caspase-3 assay) | PE | Cell Signalling Technology, London, UK | 5742 |
