# Supplementary material for: Interrogation of novel CDK2/9 inhibitor fadraciclib (CYC065) as a potential therapeutic approach for AML
Source: Cell Death Discov. 2021 Jun 10;7:137. doi: 10.1038/s41420-021-00496-y (PMC8192769; doi:10.1038/s41420-021-00496-y)
Supplement: Supplementary file 5 — Supplementary Table S4 [file 41420_2021_496_MOESM5_ESM.docx]

**Supplementary Table S4. Genetic lesions of primary human AML samples classified by their functions and their responsive status to the combination of fadraciclib with VEN, AraC, or AZA**

| **Sample** | **Karyotype** | **Genetic lesions** | | | | | | |  | **Synergy status** | | |
| --- | --- | --- | --- | --- | --- | --- | --- | --- | --- | --- | --- | --- |
|  |  | **Signalling genes** | **DNA methylation-related genes** | **Chromatin-modifying genes** | **Nucleo- phosmin gene** | **Cohesin complex genes** | **Spliceosome complex genes** | **Transcrip- tion factor fusions** |  | **FAD 2/**  **VEN 2** | **FAD 2/**  **AraC 2** | **FAD 2/**  **AZA 2** |
| AML H002 | Normal |  | *IDH2* | *KMT2A*-PTD, *ASXL1* |  |  |  |  |  | Moderate | Moderate | Marked |
| AML 44 | Monosomy 7 | *FLT3*-ITD | *DNMT3A*,  *IDH2* |  |  |  |  |  |  | Marked | Moderate | Marked |
| AML 52 | Normal | *FLT3*-ITD | *DNMT3A* |  | *NPM1* | *RAD21* |  |  |  | Slight/ Moderate | Slight | Moderate/ Marked |
| AML 31 | Complex | *KIT* |  |  | *NPM1* |  |  |  |  | Slight | Slight | Slight |
| AML 36 | Trisomy 11 |  | *DNMT3A*,  *IDH1* |  |  | *STAG2* | *SRSF2* |  |  | Slight | Slight | Slight |
| AML 22 | Normal | *FLT3*-ITD | *DNMT3A*,  *TET2* | *KMT2A*-PTD |  |  | *SRSF2* | *RUNX1* |  | Slight | Slight | Slight |
