# Supplementary figures and images for: Interrogation of novel CDK2/9 inhibitor fadraciclib (CYC065) as a potential therapeutic approach for AML
Source: Cell Death Discov. 2021 Jun 10;7:137. doi: 10.1038/s41420-021-00496-y (PMC8192769; doi:10.1038/s41420-021-00496-y)

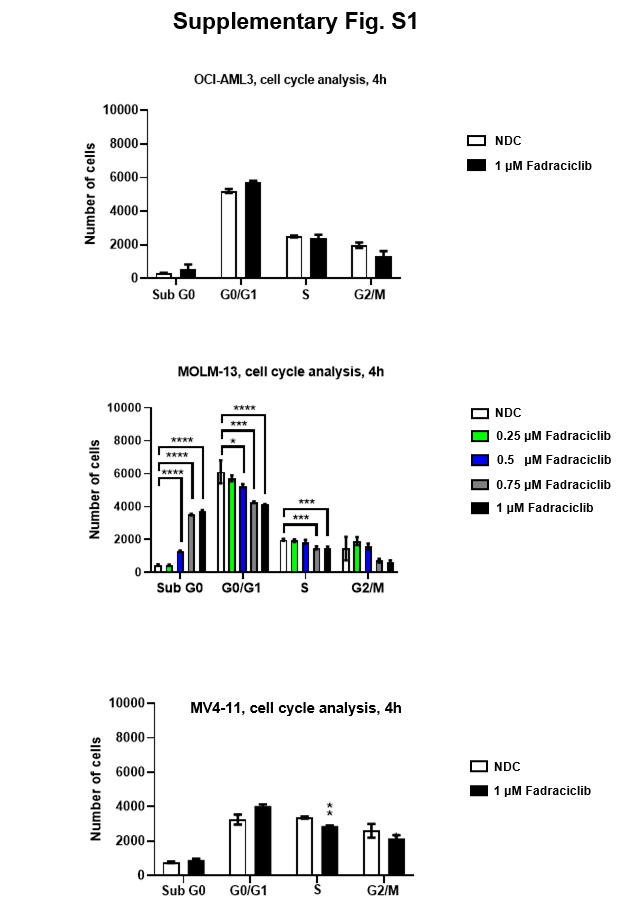

Supplement: Supplementary file 6 — Supplementary Fig. S1 [file 41420_2021_496_MOESM6_ESM.png]

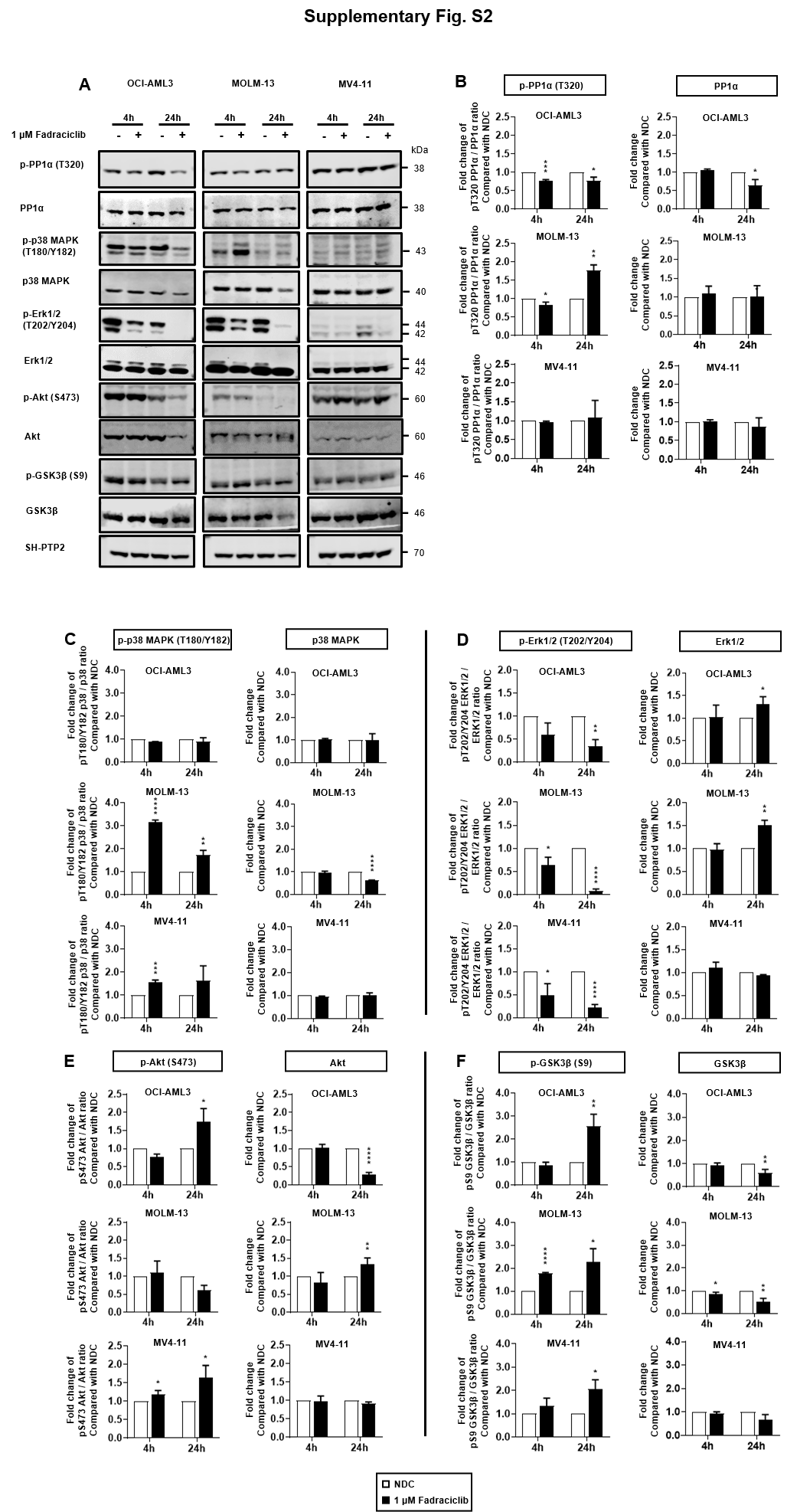

Supplement: Supplementary file 7 — Supplementary Fig. S2 [file 41420_2021_496_MOESM7_ESM.png]

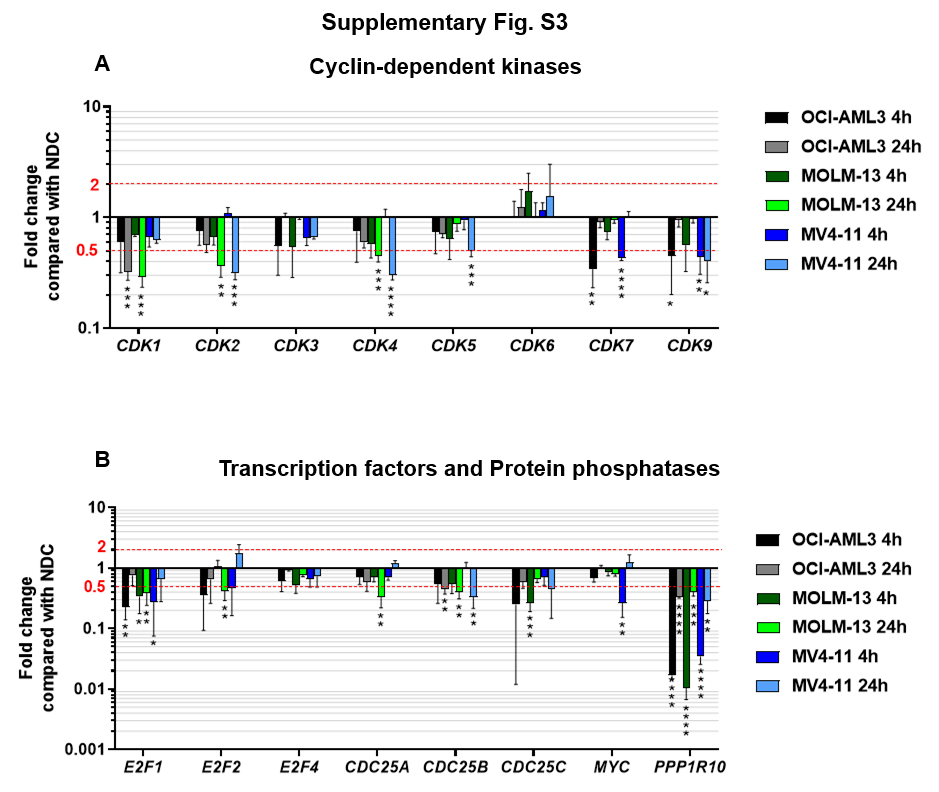

Supplement: Supplementary file 8 — Supplementary Fig. S3 [file 41420_2021_496_MOESM8_ESM.png]

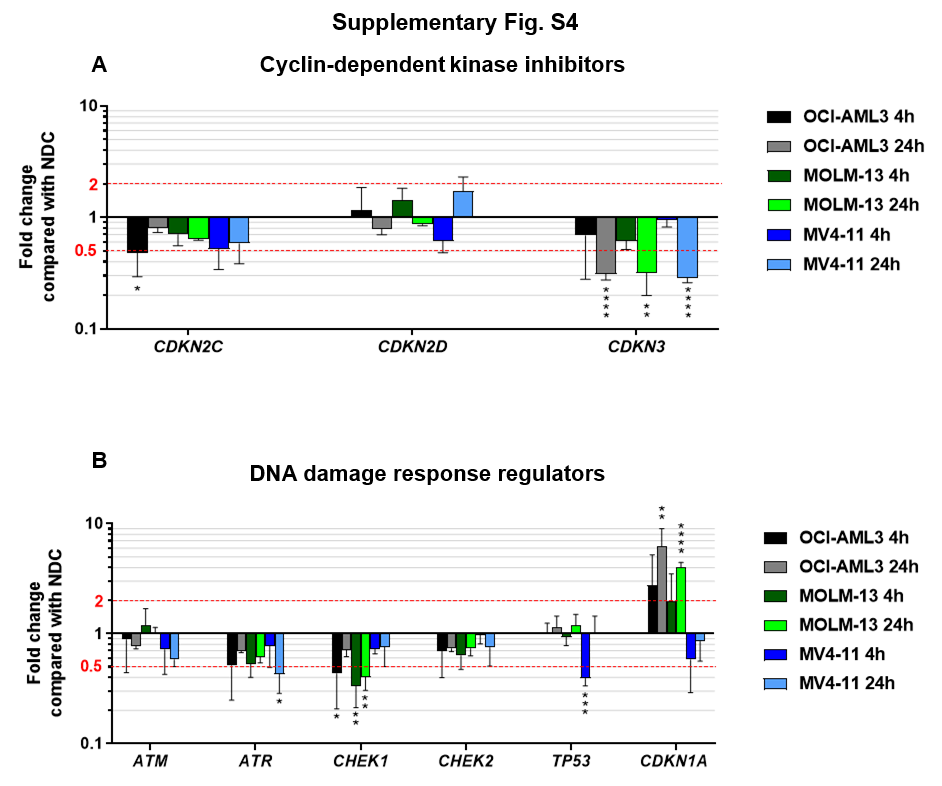

Supplement: Supplementary file 9 — Supplementary Fig. S4 [file 41420_2021_496_MOESM9_ESM.png]

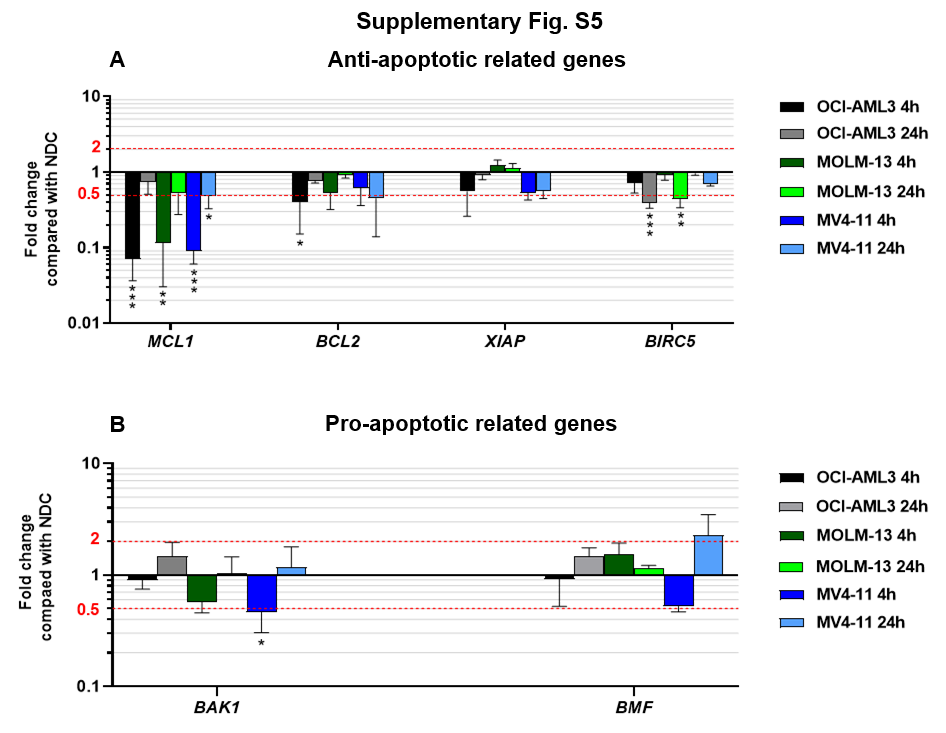

Supplement: Supplementary file 10 — Supplementary Fig. S5 [file 41420_2021_496_MOESM10_ESM.png]

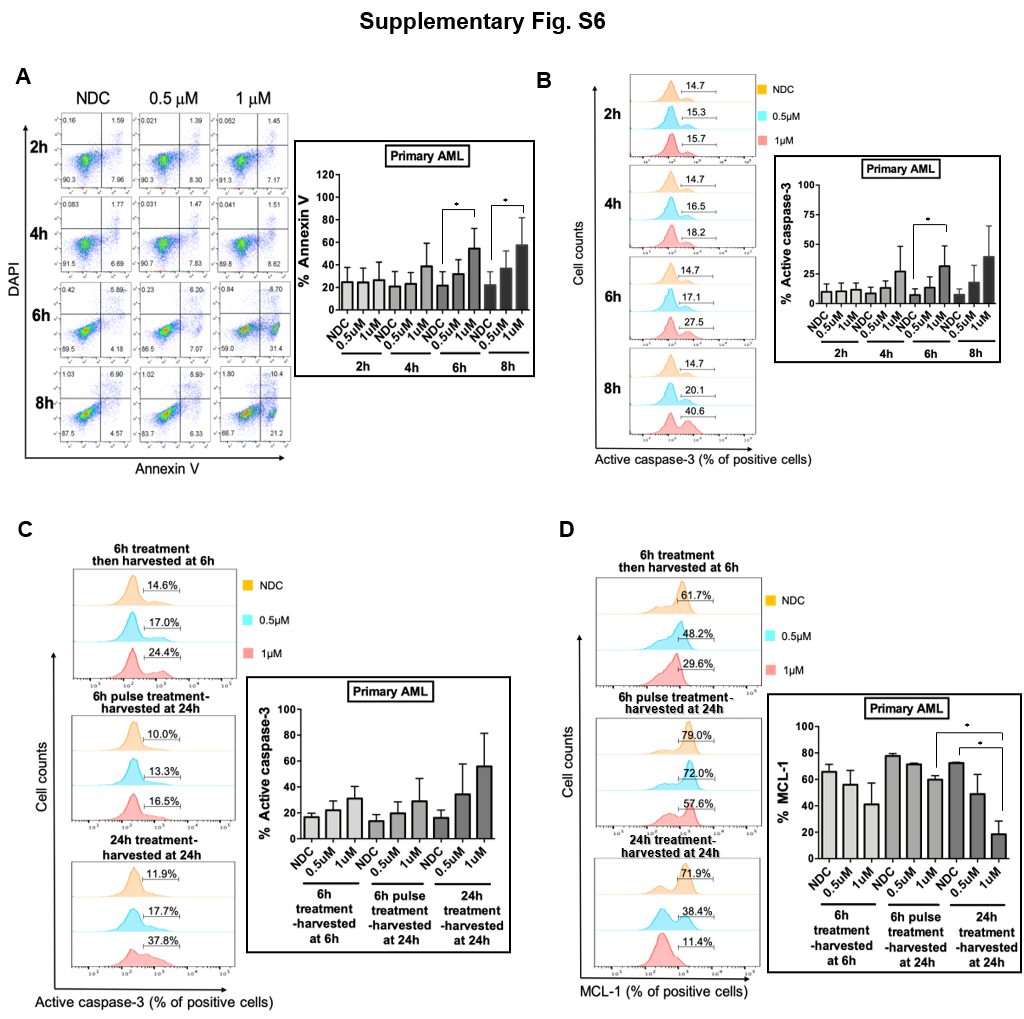

Supplement: Supplementary file 11 — Supplementary Fig. S6 [file 41420_2021_496_MOESM11_ESM.png]

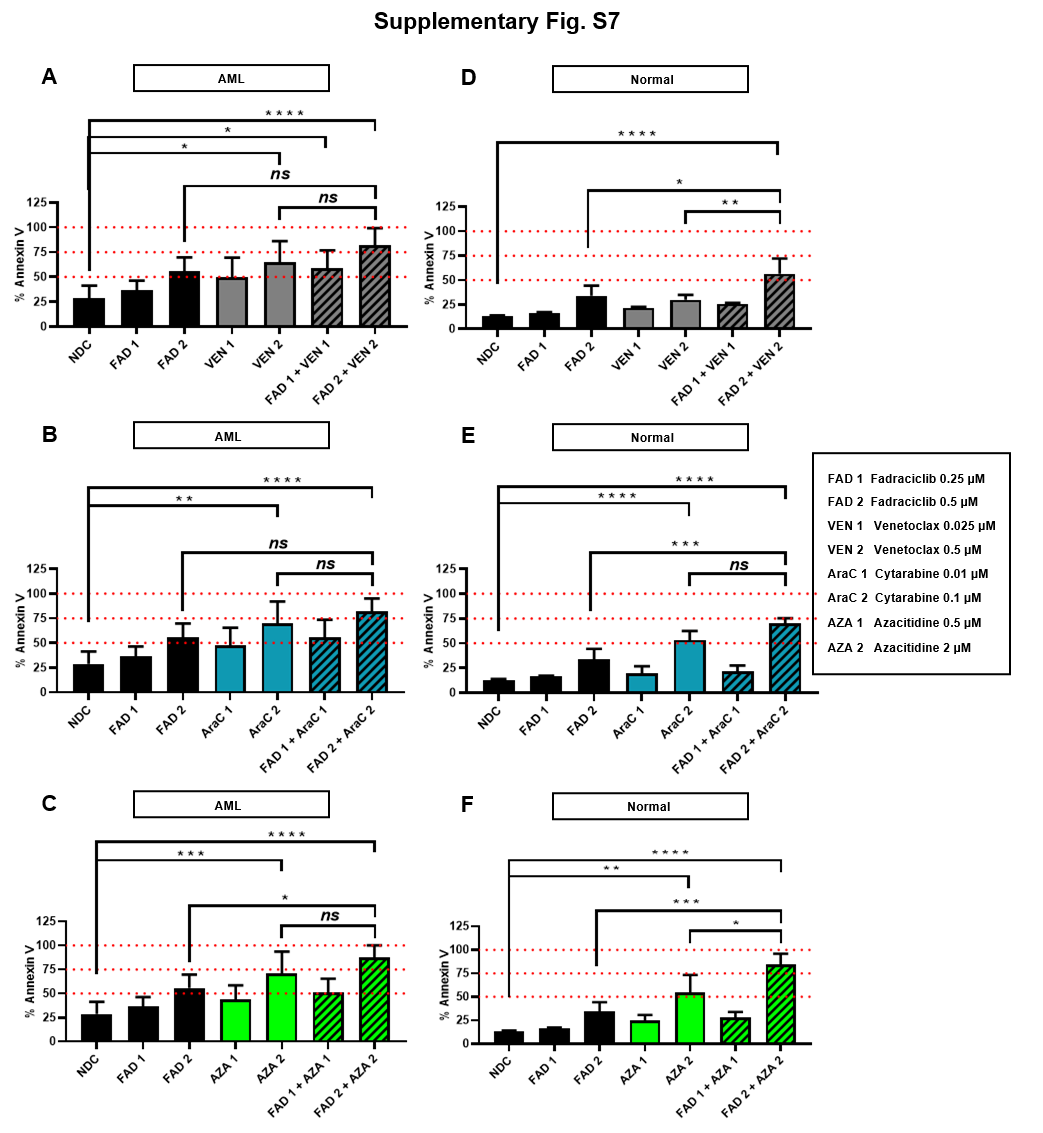

Supplement: Supplementary file 12 — Supplementary Fig. S7 [file 41420_2021_496_MOESM12_ESM.png]

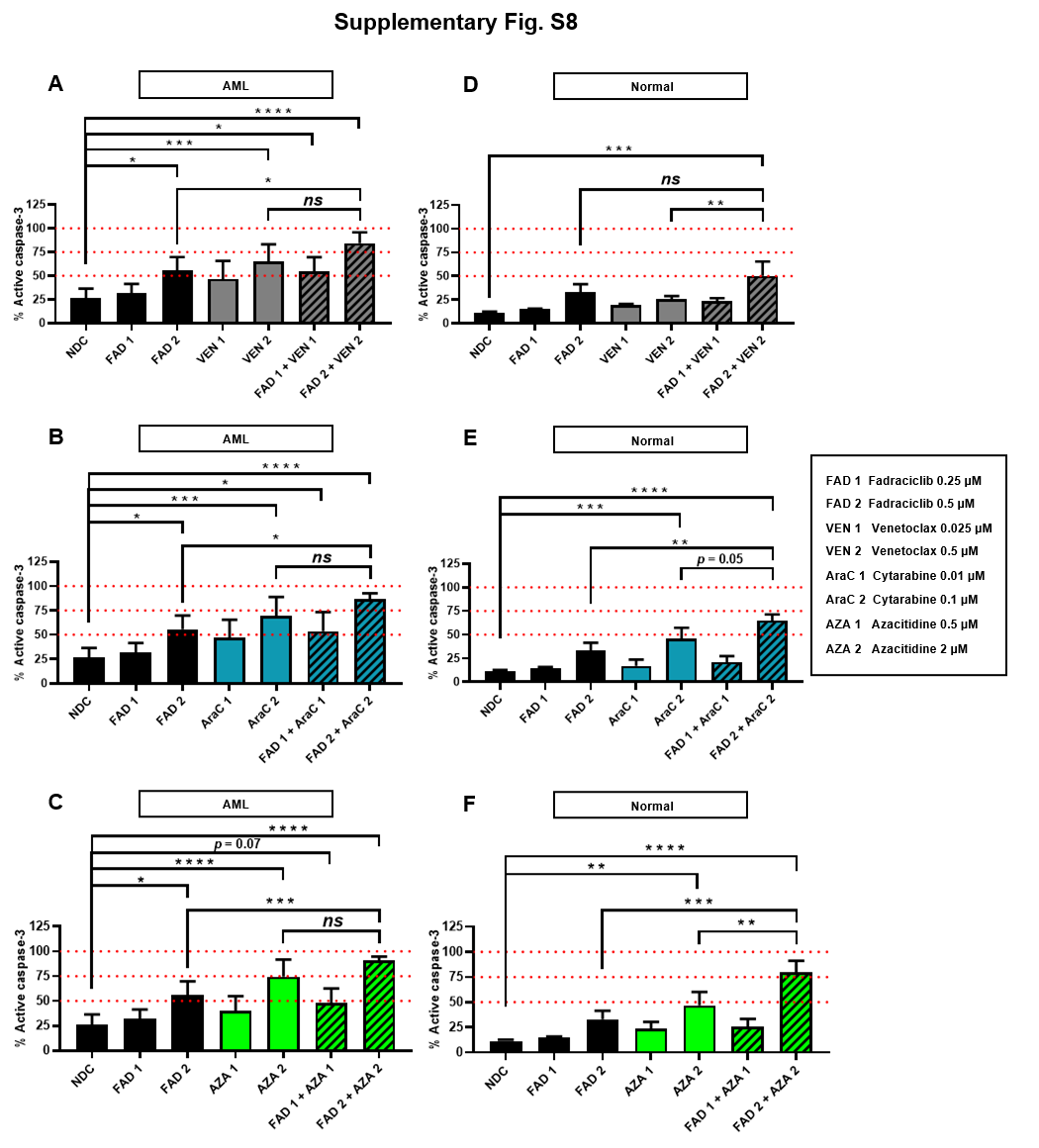

Supplement: Supplementary file 13 — Supplementary Fig. S8 [file 41420_2021_496_MOESM13_ESM.png]

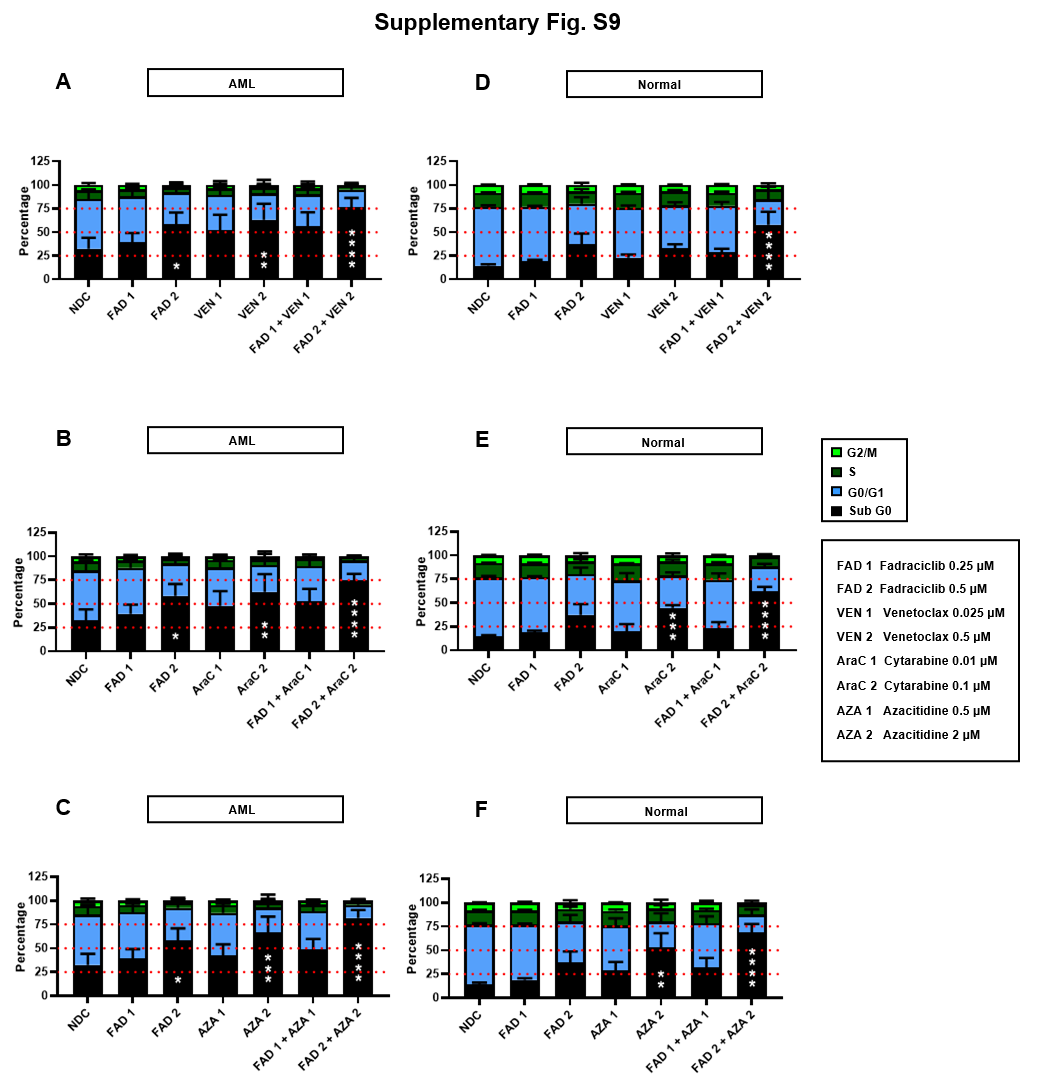

Supplement: Supplementary file 14 — Supplementary Fig. S9 [file 41420_2021_496_MOESM14_ESM.png]

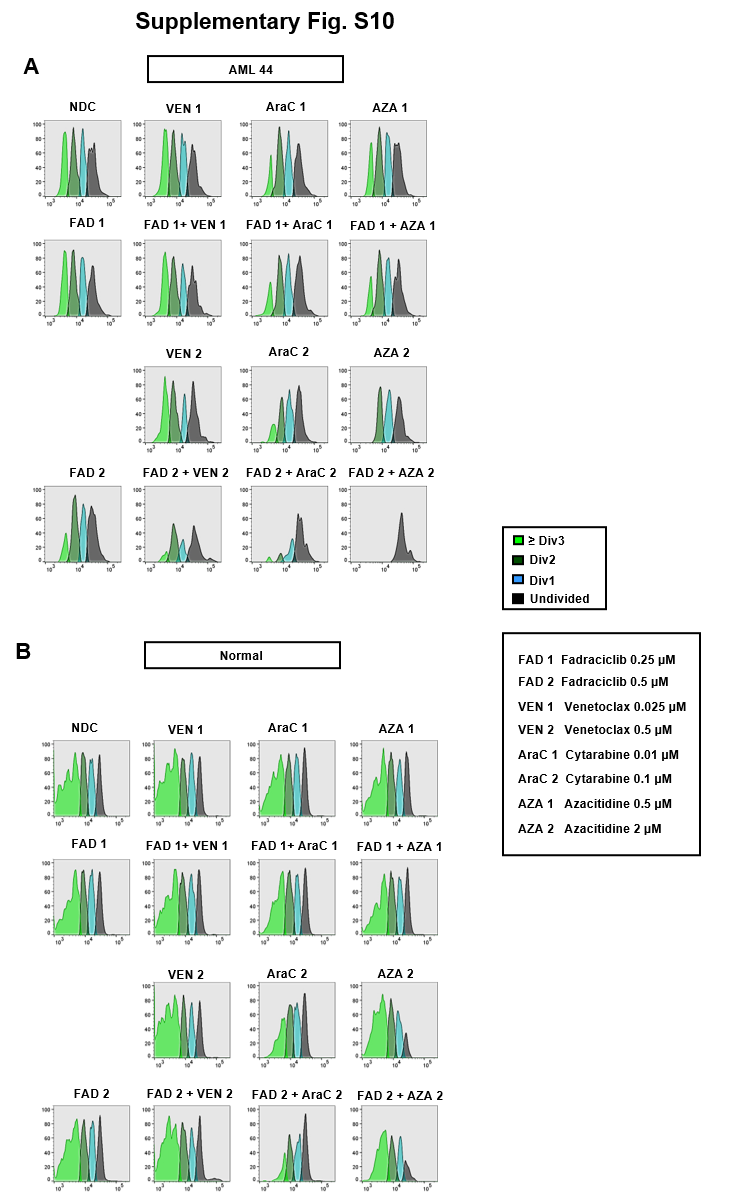

Supplement: Supplementary file 15 — Supplementary Fig. S10 [file 41420_2021_496_MOESM15_ESM.png]
